# Aberrant glial activation and synaptic defects in CaMKIIα-iCre and nestin-Cre transgenic mouse models

**DOI:** 10.1038/s41598-022-26671-4

**Published:** 2022-12-21

**Authors:** Alia O. Alia, Sohee Jeon, Jelena Popovic, Miranda A. Salvo, Katherine R. Sadleir, Robert Vassar, Leah K. Cuddy

**Affiliations:** 1 The Ken and Ruth Davee Department of Neurology, Northwestern University Feinberg School of Medicine, Chicago, IL 60611 USA; 2 Mesulam Center for Cognitive Neurology and Alzheimer’s Disease, Northwestern University Feinberg School of Medicine, Chicago, IL 60611 USA

**Keywords:** Neuroscience, Animal disease models, Genetic models

## Abstract

Current scientific research is driven by the ability to manipulate gene expression by utilizing the Cre/loxP system in transgenic mouse models. However, artifacts in Cre-driver mouse lines that introduce undesired effects and confound results are increasingly being reported. Here, we show aberrant neuroinflammation and synaptic changes in two widely used Cre-driver mouse models. Neuroinflammation in CaMKIIα-iCre mice was characterized by the activation and proliferation of microglia and astrocytes in synaptic layers of the hippocampus. Increased GFAP and Iba1 levels were observed in hippocampal brain regions of 4-, 8- and 22-month-old CaMKIIα-iCre mice compared to WT littermates. Synaptic changes in NMDAR, AMPAR, PSD95 and phosphorylated CaMKIIα became apparent in 8-month-old CaMKIIα-iCre mice but were not observed in 4-month-old CaMKIIα-iCre mice. Synaptophysin and synaptoporin were unchanged in CaMKIIα-iCre compared to WT mice, suggesting that synaptic alterations may occur in excitatory postsynaptic regions in which iCre is predominantly expressed. Finally, hippocampal volume was reduced in 22-month-old CaMKIIα-iCre mice compared to WT mice. We tested the brains of mice of additional common Cre-driver mouse models for neuroinflammation; the nestin-Cre mouse model showed synaptic changes and astrocytosis marked by increased GFAP+ astrocytes in cortical and hippocampal regions, while the original CaMKIIα-Cre T29-1 strain was comparable to WT mice. The mechanisms underlying abnormal neuroinflammation in nestin-Cre and CaMKIIα-iCre are unknown but may be associated with high levels of Cre expression. Our findings are critical to the scientific community and demonstrate that the correct Cre-driver controls must be included in all studies using these mice.

## Introduction

In the field of biomedical research, genetic engineering is an essential technology to investigate protein function in specific tissues and cell types. The Cre/loxP system is an advanced method of gene targeting that allows for precise control over tissue specificity and timing of gene expression in mice, while avoiding complications that arise during constitutive gene knockout (KO) such as early embryonic lethality. In the Cre/loxP system, Cre recombinase, a prokaryotic enzyme isolated from the P1-bacteriophage, is expressed in genetically engineered mice under the control of a tissue-specific promoter and catalyzes the recombination between two loxP sites flanking an exon of a gene of interest^1–3^. Mice with LoxP sites flanking a gene of interest (floxed) are bred to transgenic mice expressing Cre to create gene KO and knock-in (KI) mice^4,5^. Many Cre-driver mice are available that express Cre in diverse patterns within the central nervous system (CNS) to investigate genes of interest within specific regions and cell types of the brain^6–8^. The selection of the promoter driving Cre expression is critical, as this controls the timing and location of Cre activity and thus of gene KO.

The calcium/calmodulin-dependent protein kinase II alpha subunit (CaMKII*α*) promoter is routinely used to drive Cre expression in excitatory forebrain neurons of the CNS. Numerous strains of CaMKII*α* Cre-driver mice have been developed^9^, with the most widely used strains being the original CaMKII*α* Cre-driver mouse, the CaMKII*α* Cre T29-1 strain^10^ and the CaMKIIα-iCre strain^11^. CaMKII*α* is a member of the CaMKII family of serine/threonine protein kinases activated by Ca^2+^ and is endogenously expressed within pyramidal cells of the hippocampus and the cerebral cortex^12,13^. Within the hippocampus, CaMKII*α* is strongly expressed in the pyramidal CA1 cell layers and in molecular layers containing dendritic spines^14,15^. Therefore, these mice are often used in studies of neurological disorders, memory and learning, and synaptic plasticity^9^.

CaMKII*α* Cre-drivers are typically generated using small plasmid-based expression vectors carrying CaMKII*α* and prokaryotic Cre, including the CaMKII*α* Cre T29-1 mouse. However, the CaMKIIα-iCre mouse is distinct in that it was developed using a bacterial artificial chromosome (BAC), to express iCre^11^. An advantage of this method is that all genetic components required to recapitulate endogenous CaMKIIα gene expression are expressed within the BAC. The BAC also harbors improved Cre recombinase (iCre), a genetically modified version of Cre optimized for maximal Cre expression in mammals^11,16^. Thus, CaMKIIα-iCre mice express higher levels of Cre resulting in more accurate and efficient gene knockout, and to date, this CaMKII*α* Cre-driver mouse has been published in 146 studies^9^.

The spatiotemporal control of gene expression by tissue specific promoters is a clear advantage of the Cre/loxP system over traditional constitutive gene KO approaches, yet serious caveats of Cre-driver mice have been reported^17,18^. For example, a recent meta-analysis indicated that germline recombination is present in 64.1% of 64 commonly used strains of Cre-drivers for CNS gene KO^19^. Additionally, toxicity from Cre itself has been reported. Cre expression in neural progenitors causes hydrocephalus, microencephaly and metabolic disturbances in nestin-Cre mice^20–23^ and induces apoptosis and reduces proliferation in cultured cell lines^24^. Cre-driver mice are commonly generated through pronuclear microinjection of the Cre transgene, so the random insertion of the Cre transgene could disrupt endogenous genes and lead to off-target effects in strains of Cre-driver mice. The disruption of endogenous gene expression due to the copy number and integration site of the BAC iCre transgene has been reported to cause ectopic overexpression of the Ca^2+^ sensing protein synaptotagmin 2 in excitatory presynaptic regions in CaMKIIα-iCre mice^25^. The overexpression of synaptotagmin 2 led to alterations in cerebral synaptic activity in CaMKIIα-iCre mice, suggested by hyperactivity and enhanced learning in fear conditioning tests^25^.

In this study we report abnormal activation of microglia and astrocytes and altered expression of synaptic proteins in two commonly used strains of Cre-driver mice. We found reactive microglia and astrocytes throughout the pyramidal, stratum oriens (SO) and stratum radiatum (SR) hippocampal CA1 layers of CaMKIIα-iCre mice^11^. We assessed additional Cre-drivers, including CaMKII*α* Cre T29-1 and nestin-Cre^21^ strains, and found astrocytosis and synaptic alterations in hippocampal and cortical brain regions of nestin-Cre mice, which highly express Cre in neural progenitors. CaMKII*α* Cre T29-1 express low levels of Cre and were comparable to WT mice. The mechanisms causing glial activation in nestin-Cre and CaMKIIα-iCre mice are unknown, although our results suggest that high levels of Cre expression in neurons may activate microglia and astrocytes and lead to changes in synaptic protein expression. Typical Cre-driver breeding strategies wherein Cre-driver mice are crossed to floxed allele mice exclude Cre-driver-only controls from experimental design, raising the question of whether conclusions can be drawn from past publications using floxed allele-only mice as controls. Our results agree with studies showing that adverse phenotypes can arise in commonly used Cre-driver mice and indicate that nestin-Cre and CaMKIIα-iCre mice should only be used if the appropriate Cre-driver-only control is included, as these unexpected phenotypes must be taken into consideration.

## Materials and methods

### Study design

Cre-expressing mice are typically bred in a breeding scheme wherein Cre-driver mice are crossed to floxed allele mice to create gene KO, KI or reporter mice. In this study, we find that in the absence of floxed alleles, activated microglia and astrocytes proliferated in a distinct pattern throughout the SR and SO hippocampal layers of CaMKIIα-iCre mice compared to WT littermates. CaMKIIα-iCre mice were analyzed at 4, 8 and 22-months of age and an equivalent number of male and female mice were analyzed for all parameters. The figure legends have specific n values and females are represented as circles and males as triangles in all graphs. Synaptic changes and hippocampal volume loss were analyzed in aged CaMKIIα-iCre mice. We initially observed an aberrant neuroinflammation in the hippocampus of CaMKIIα-iCre mice^11^, and we determined the presence of Cre-associated neuroinflammation in two additional strains of Cre recombinase expressing mice, the CaMKIIα-Cre T29-1 strain^10^ and the nestin-Cre mouse model^21^. These mice were analyzed at 4-months-old. By analyzing these additional mice and WT controls, we established that neuroinflammation and synapse changes revealed in CaMKIIα-iCre were likely caused by Cre recombinase expression. We investigated these effects by utilizing immunofluorescence markers that demonstrate various states of activity, reactivity, and formation of glial cells within areas of the hippocampus that govern synaptic plasticity and function in learning and memory, as well as the quantification of these cell markers. The analysis of these neuronal and synaptic markers highlighted the phenotypic neuroinflammatory effect of Cre on presynaptic and postsynaptic regions within specific areas of the brain. We further confirmed the results of immunofluorescence through western blot analysis by means of probing for neuronal inflammatory and synaptic markers and the quantification of those results.

### Ethics statement

All experimental protocols were approved by the Northwestern University Institutional Animal Care and Use Committee (IACUC). All methods were carried out in accordance with relevant guidelines and regulations. All methods reported are in accordance with ARRIVE guidelines.

### Animals

Animals were housed and monitored in accordance with IACUC. Mice were maintained on a 12:12 light: dark cycle and consumed a standard rodent diet and water ad libitum. CaMKIIα-iCre mice were a generous gift of Dr. Warren Tourtellotte (Cedars-Sinai) and bred to C57BL/6J mice and maintained as heterozygotes. Homozygous CaMKIIα-Cre T29-1 mice were purchased from The Jackson Laboratory (strain #005359) and crossed with C57BL/6J mice to produce heterozygous mice for analysis. Homozygous Nestin-Cre mice were purchased from The Jackson Laboratory (strain #003771) and crossed with C57BL/6J mice to produce heterozygous mice. Wild-type C57BL/6J littermates were used as controls for all groups in this study. All animals analyzed were included in statistical analysis. To confirm genotypes, all mice used in the study were PCR-genotyped in house for Cre via primers purchased from Integrated DNA Technologies (IDT) and used in accordance with Jackson Laboratory standard genotyping protocols. The CaMKIIα-iCre genotyping protocol involved a two primer reaction, the forward DNA oligo consisted of nineteen bases 5ʹ-AGAAGCCCCAAGCTCGTCA-3ʹ, and the reverse DNA oligo consisted of eighteen bases 5ʹ-CAGCAGGGAACCATTTCC-3ʹ. The Nestin-Cre genotyping protocol involved a three primer reaction, the forward DNA oligo consisted of twenty-one bases 5ʹ-CCTTCCTGAAGCAGTAGAGCA-3ʹ, the reverse DNA oligo consisted of twenty bases 5ʹ-GCCTTATTGTGGAAGGACTG-3ʹ, and the wildtype forward consisted of twenty-one bases 5ʹ-TTGCTAAAGCGCTACATAGGA-3ʹ. The CaMKIIα-Cre T29-1 strain genotyping protocol involved a four primer reaction, the forward DNA oligo consisted of twenty bases 5ʹ-CGTCCATCTGGTCAGAAAAG-3ʹ, the reverse DNA oligo consisted of twenty bases 5ʹ-TCTTCTTCTTGGGCATGGTC-3ʹ, the internal positive control forward consisted of twenty bases 5ʹ-AGTGGCCTCTTCCAGAAATG-3ʹ, and the internal positive control reverse DNA oligo consisted of twenty bases 5ʹ-TGCGACTGTGTCTGATTTCC-3ʹ.

### Tissue extraction and preparation

Animals were euthanized by intraperitoneal injection of xylazine (15 mg/kg) and ketamine (100 mg/kg) followed by transcardial perfusion with 10 ml 1× cold phosphate-buffer saline (PBS) containing phenylmethylsulfonyl fluoride (20 mg/ml in EtOH) 1:1000, dithiothreitol (1 M) 1:10,000, leupeptin (5 mg/ml) 1:10,000 and sodium orthovanadate (200 mM) 1:10,000. Mouse brains were extracted immediately following perfusion and right hemibrains were dissected on ice into two regions: cortex and hippocampus. The tissue was flash frozen in liquid nitrogen and stored at − 80 °C until biochemical analysis.

All tissue lysis buffers contained Halt phosphatase inhibitor (#78420, Thermo Fisher Scientific) and protease inhibitor cocktail III (#535140, Millipore). Hippocampus and cortex tissues were weighed and homogenized with a dounce homogenizer in a 1:10 (w/v) 1× PBS, then centrifuged at 4 °C at 14,000 RPM for thirty minutes. The supernatant was removed as the soluble fraction and stored at − 80 °C. The remaining pellet was extracted by the addition of radioimmuprecipitation assay buffer (RIPA) (50 mM tris, 0.15 M NaCl, 1% octylphenoxypolyethoxyethanol (IGEPAL), 1 mM EDTA, 1 mM EGTA, 0.1% SDS, 0.5% sodium deoxylate at pH 8) at 1:10 (w/v). The samples were sonicated for twenty seconds on ice, followed by centrifugation at 14,000 RPM for 30 min at 4 °C. The supernatant was removed and a bicinchoninic assay (BCA) (#23225, Thermo Fisher Scientific) was performed to measure protein concentration. After determining protein concentration, the appropriate volumes of samples and buffers were calculated to reach the same final protein concentration (1 mg/ml). NuPAGE LDS Sample Buffer (4×) (#NP0008, Thermo Fisher Scientific) with 4% β-Mercaptoethanol (#M6250, Millipore Sigma) was added to all samples followed by heating at 95 °C for 10 min on a heat block.

### Immunoblotting

Equal amounts of protein homogenates (20ug) were loaded into NuPAGE midi Bis–tris gels (#WG1403BOX, Thermo Fisher Scientific) set up in Criterion Cell electrophoresis chambers (Bio-Rad) containing 1× MOPS running buffer using 50 mM MOPS (#PHG0007, Millipore Sigma) prepared in 50 mM Tris Base (#DST60040-10000, Dot Scientific), 1 mM EDTA (#50-841-667, Teknova) and 0.1% SDS (#50-751-6948, Quality Scientific). Gels ran at approximately 140–160 V for 1–1.5 h, or until the samples reached the bottom of the gel. Gels were transferred to nitrocellulose membranes in 5× transfer buffer (Bio-rad) and ran for 45 min at a current of 1.1A using the Trans-Blot Turbo Transfer system (Bio-rad). The membrane was washed 3 times in washing buffer (1× PBS, 0.1% TWEEN-20) for 5 min, then blocked in SuperBlock blocking buffer blotting in PBS (#37517, Thermo Fisher Scientific) at room temperature for 1 h. Primary antibodies were prepared in washing buffer with 10% SuperBlock blocking buffer then added to the membranes and allowed to incubate overnight in 4 °C. Primary antibody was then removed, and the membranes were washed in washing buffer 3 times for 5 min at room temperature. Secondary antibodies (horse anti-mouse, #P1-2000 Vector Labs, goat anti-rabbit, #P1-1000 Vector Labs) were prepared in wash buffer with 10% SuperBlock and added to the membranes at 1:10,000. The membranes were incubated in secondary antibodies for 60 min and then washed 3 times for 5 min in washing buffer. The blots were developed using SuperSignal West Femto Maximum Sensitivity Substrate (#34096, Thermo Fisher Scientific) and SuperSignal West Pico PLUS Chemiluminecsent Substrate (#34580, Thermo Fisher Scientific) and imaged on a ProteinSimple FCR imager. Chemiluminescent signals were quantified using AlphaView software (ProteinSimple).

### Immunohistochemistry

Left hemibrains were immersion-fixed in 10% formalin and kept in 30% sucrose 1× PBS solution at 4 °C for long-term storage. Hemibrains were mounted coronally and serially sectioned into 12-well plates at 30 microns thick using a freezing-sliding microtome and preserved in cryoprotective solution (1× PBS, 30% sucrose and 30% ethylene glycol). Brain sections were washed in 1× tris-buffer saline (TBS) 3 times for 5 min on an orbital shaker at 180 RPM. Sections were transferred to a glycine solution (1XTBS, 0.25% triton X-100, 16 mM glycine) and incubated on an orbital shaker at 180 RPM for 1 h at room temperature. Brain sections were washed again 3 times in 1× TBS for 5 min. Sections were then transferred to blocking solution (1× TBS, 0.25% triton X-100, 5% donkey serum) and allowed to incubate for a duration of 2 h on an orbital shaker at 180RPM. Sections were then washed in 1× TBS, 0.25% triton X-100 and 1% BSA 2 times for 10 min. Primary antibodies were prepared in 1× TBS, 0.25% triton X-100 and 1% BSA, and incubated overnight at 4 °C on an orbital shaker at 110 RPM. The sections were then washed again in 1× TBS, 0.25% triton X-100 and 1% BSA 3 times for 10 min on an orbital shaker at 180 RPM until they were transferred to Alexa fluor-labeled secondary antibodies (Invitrogen) at a dilution of 1:1000 prepared in 1XTBS, 0.25% triton X-100 and 1% BSA and allowed to shake for 2 h at 180 RPM. All sections were washed 3 times for 15 min in 1XTBS and then mounted onto slides using ProLong Gold (#P36934, Thermo Fisher Scientific) and imaged on a Nikon A1 laser scanning confocal microscope or a Nikon Ti2 wide-field microscope (Northwestern University Center for Advanced Microscopy).

### Image quantification

To measure the volume of the hippocampus, brains were serially sectioned into 30 μm thick sections in a 12-well plate. Sequential sections chosen for analysis were from Bregma coordinates of approximately − 0.94 to − 3.40 mm through the hippocampus (eight sections per brain, 360 μm apart) and areas of interest were traced manually and measured using ImageJ software. Sections were immunostained as described above using anti-NeuN primary antibody and DAPI (4′,6-diamidino-2-phenylindole), and then imaged with a 4× objective using a Ti2 wide-field microscope. Volume was calculated using the following formula: volume = (sum of area) × 0.36 mm. For immunofluorescence quantification of Iba1, GFAP, synaptoporin and NeuN-covered area, three to four coronal sections from Bregma coordinates of about − 1.70 to − 3.52 mm were obtained. Brain regions were immunostained using anti-Iba1, anti-GFAP, anti-synaptoporin and anti-NeuN primary antibodies as described above, and images were captured using a 10× objective and a Ti2 wide-field microscope. Nikon NIS-Elements Software (Northwestern University Nikon Imaging Centre) was used to set intensity and size thresholds to eliminate background staining. To calculate Iba1, GFAP, synaptoporin and NeuN- covered area within the hippocampus or cortex, SR, SO or pyramidal layers, each area was traced manually using NIS-Elements, and a binary channel was created for each region of interest. The average of three sections was obtained to calculate the covered area in the SR, SO, pyramidal layers, hippocampus and cortex for each signal in each mouse. All imaging, section selection, tracing, and volume analysis were performed by someone blind to the genotypes of the animals.

### Statistical analyses

Statistical tests were performed using GraphPad Prism software v7.05 (http://graphpad.com/scientific-software/prism/). Detailed test information including the specific statistical tests, number of replicates, and *P* values are stated in the figure or the figure legend. For all quantifications, the data are plotted as the mean ± SEM. Significance was concluded when *P* < 0.05, indicated by *P < 0.05, **P < 0.01, ***P < 0.001, and ****P < 0.0001.

## Results

### Activation and proliferation of microglia and astrocytes in the stratum oriens and radiatum in the hippocampus of CaMKIIα-iCre mice

While investigating the effect of gene knockout on glial cell activity in floxed allele CaMKIIα-iCre mice, we incidentally observed abnormal neuroinflammation in hippocampal brain regions of 8-month-old CaMKIIα-iCre mice that do not express floxed alleles. Therefore, we sought to investigate and characterize this abnormal phenotype in CaMKIIα-iCre mice in this study.

**Figure 1:**
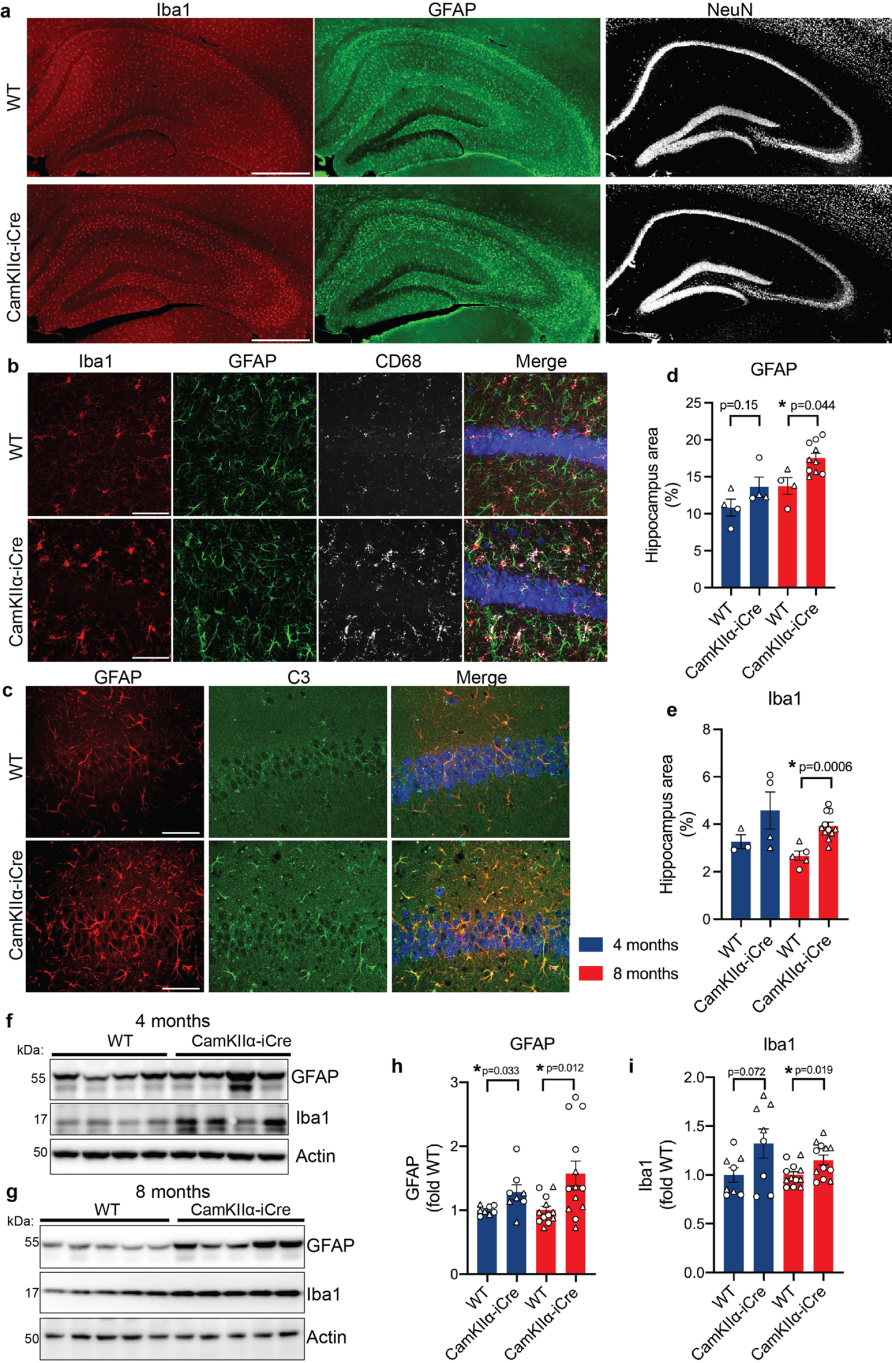
Proliferation and activation of microglia and astrocytes in the pyramidal, stratum oriens and radiatum hippocampal layers of CaMKIIα-iCre mice. **(A)** Immunofluorescence microscopy of representative coronal brain sections showing the hippocampus from female 8-month-old WT and CaMKIIα-iCre mice labeled for Iba1 (red), GFAP (green) and NeuN (white). Scale bar, 500 µm. Note the accumulation of Iba1-positive microglia and GFAP-positive astrocytes in the SO, SR and pyramidal layers of the hippocampus of CaMKIIα-iCre mice. **(B)** High magnification confocal microscopy images of the CA1 hippocampal region showing Iba1 (red), GFAP (green), CD68 (white) and NeuN (blue) immunostaining in female 8-month-old WT and CaMKIIα-iCre mice. Scale bar, 50 µm. **(C)** High magnification confocal microscopy images of the CA1 hippocampal region showing GFAP (red), C3 (green) and NeuN (blue) immunostaining in female 8-month-old WT and CaMKIIα-iCre mice. Scale bar, 50 µm. Quantification of GFAP **(D)** or Iba1 **(E)** percentage of area covering the hippocampus from 4-month-old and 8-month-old WT and CaMKIIα-iCre mice (WT, n = 4–5; CaMKIIα-iCre, n = 10–11). Immunoblot of hippocampal homogenates from 4-month-old **(F)** and 8-month-old **(G)** female WT and CaMKIIα-iCre mice probed for GFAP, Iba1 and actin. Original immunoblots are shown in Fig. S3A,B. Quantification of GFAP **(H)** and Iba1 **(I)** immunoblots in **(F,G)** normalized to actin and expressed as fold of WT (4-month-old WT, n = 8; 4-month-old CaMKIIα-iCre, n = 8; 8-month-old WT, n = 12; 8-month-old CaMKIIα-iCre, n = 13). Circles = females, triangles = males. Unpaired t-test was performed for all quantifications and values are mean ± SEM.

Significant increases in microglia marker Iba1 and astrocytic protein GFAP in the hippocampus were observed in 8-month-old CaMKIIα-iCre mice compared to wild-type (WT) littermates of the same genetic background (C57/BL6) (Fig. 1A,D,E). We also detected increased expression of CD68, which is highly expressed during microglia activation^26^ and C3, which is expressed by reactive astrocytes^27^ (Fig. 1B,C). We assessed CaMKIIα-iCre and WT mice at 4-months of age and found trends toward an increase in Iba1 and GFAP to levels comparable to 8-months in CaMKIIα-iCre mice (Fig. 1D,E). The activation and proliferation of microglia and astrocytes observed by immunofluorescence microscopy was confirmed by performing immunoblot analysis for GFAP and Iba1 in hippocampal homogenates of 4- and 8-months old WT and CaMKIIα-iCre mice (Fig. 1F,G). Consistently, at 4 and 8 months, Iba1 and GFAP were increased to comparable levels in CaMKIIα-iCre mice (Fig. 1H,I). To confirm our findings, we also assessed Iba1 and GFAP levels in CaMKIIα-iCre mice that were bred and housed in a separate facility. Here we found increased levels of Iba1 and GFAP in hippocampal homogenates of CaMKIIα-iCre mice compared to WT mice at 2 and 5 months of age (Fig. S1A).

In the hippocampus of CaMKIIα-iCre mice, activated microglia and astrocytes appeared to proliferate and migrate into the pyramidal, SO and SR layers that surround CA1 pyramidal neurons, compared to WT mice (Fig. 1A–C). Iba1 and GFAP covered area in hippocampal brain regions were significantly increased in 8-month-old mice (Fig. 1D–I), so we next analyzed the distribution of Iba1 and GFAP-positive glial cells across the CA1 pyramidal neurons, SR and SO layers of the hippocampus in 8-month-old WT and CaMKIIα-iCre mice. The most significant increase in GFAP and Iba1 was in the SR layers surrounding CA1 pyramidal neurons in CaMKIIα-iCre mice (Fig. S1B,C). Significant increases were also observed in the SO (Fig. S1D,E) and pyramidal layers (Fig. S1F,G). Together, elevated levels of Iba1, CD68, GFAP and C3 indicated that microglia and astrocytes were in a persistent reactive, activated state in CaMKIIα-iCre mice. These results reveal increased expression of CD68 that is highly linked to microglial activation and increased expression of C3, suggesting an upregulation of reactive astrocytes. These changes occurred in the pyramidal, SR and SO layers of the hippocampus and could subsequently result in the adaptation of a neurotoxic hippocampal phenotype that disrupts synaptic processes within the brain.

### Activation and proliferation of microglia and astrocytes in the cortex of female CaMKIIα-iCre mice

**Figure 2:**
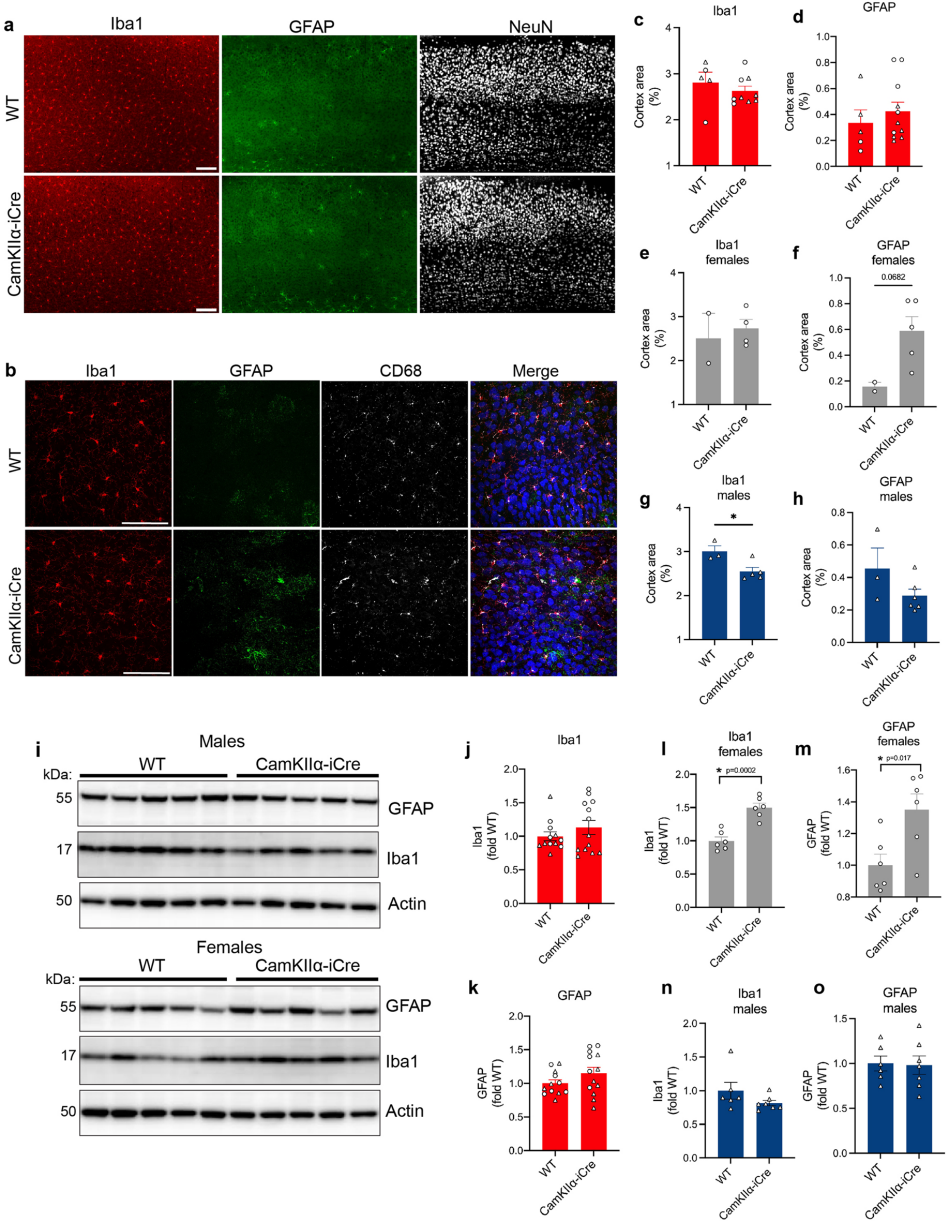
Increased expression of Iba1 and GFAP in the cortex of female CaMKIIα-iCre expressing mice. **(A)** Immunofluorescence microscopy of representative coronal brain sections showing the cortex from 8-month-old female WT and CaMKIIα-iCre mice labeled for Iba1 (red), GFAP (green) and NeuN (white). Scale bar, 100 µm. **(B)** High magnification confocal microscopy images of layer 5 cortex region in (**A)** showing Iba1 (red), GFAP (green), CD68 (white) and NeuN (blue) immunostaining. Scale bar, 100 µm. Quantification of layer 5 cortex Iba1 and GFAP immunostaining as a percentage of area covering the cortex from WT and CaMKIIα-iCre mice for males and females combined **(C,D)** and individually analyzed for females **(E,F)** and males **(G,H)** (WT, n = 5; CaMKIIα-iCre, n = 9–11). **(I)** Immunoblot of cortical homogenates from 8-month-old male (upper) and female (lower) WT and CaMKIIα-iCre mice probed for GFAP, Iba1 and actin. Original immunoblots are shown in Fig. S3C. Quantification of Iba1 **(J)** and GFAP **(K)** immunoblots in (**I**) for males and females combined and individually analyzed for females **(L,M)** and males **(N,O)** normalized to actin and expressed as fold of WT (male WT, n = 11; CaMKIIα-iCre, n = 12). Circles = females, triangles = males. Unpaired t-test was performed for all quantifications and values are mean ± SEM.

The CaMKII*α* promoter is expressed at high levels by pyramidal neurons of both the hippocampus and the cerebral cortex^11^, so we next analyzed whether the expression of iCre in pyramidal neurons of the cortex was associated with increased Iba1 and GFAP. Immunofluorescence microscopy revealed no differences in Iba1 and GFAP covered area in cortical brain regions of 8-month-old CaMKIIα-iCre mice compared to WT mice when male and female mice were analyzed together (Fig. 2A–D). However, when quantifications were performed separately, there was a trend toward an increase in Iba1 (Fig. 2E) and GFAP (Fig. 2F) covered area in female CaMKIIα-iCre mice and a decrease in Iba1 (Fig. 2G) and GFAP (Fig. 2H) covered area in male CaMKIIα-iCre mice compared to WT control mice. Immunoblot analyses revealed similar results; there were no changes in Iba1 and GFAP in cortical brain homogenates when male and female CaMKIIα-iCre and WT mice were analyzed together (Fig. 2I–K), yet significantly increased Iba1 and GFAP was observed in cortical homogenates of female (Fig. 2L,M), but not male (Fig. 2N,O), CaMKIIα-iCre mice compared to WT mice.

To determine sex differences in Iba1 and GFAP in the hippocampus, we analyzed hippocampal GFAP (Fig. 1D,H) and Iba1 (Fig. 1E,H) separately for male and female 8-month-old CaMKIIα-iCre mice and WT mice. Here, there was a trend toward an increase in GFAP (Fig. S2A,C) and a significant increase in Iba1 (Fig. S2B,D) in male CaMKIIα-iCre mice compared to WT mice. Female CaMKIIα-iCre mice showed significant increases in GFAP (Fig. S2E,G) and Iba1 (Fig. S2F,H) in CaMKIIα-iCre mice in hippocampal brain regions compared to WT control mice. Our results indicate that physiological sex differences in rodent microglia and astrocytes^28–30^ residing in cortical brain regions caused the differential results in male and female CaMKIIα-iCre.

### CaMKIIα-iCre mice show synaptic defects in the SO and SR layers of the hippocampus

Microglia and astrocytes regulate the excitatory activity of neural circuits in the hippocampus, during both development and in the adult brain, by performing activity-dependent synaptic pruning^31–33^. In CaMKIIα-iCre mice, microglia and astrocytes migrated to the pyramidal, SR and SO layers (Fig. 1A, Fig. S1B–G) that are enriched with synaptic connections of the hippocampus. Therefore, we hypothesized that activated microglia and astrocytes may engulf excitatory synapses and alter synaptic function in the CaMKIIα-iCre hippocampus.

**Figure 3:**
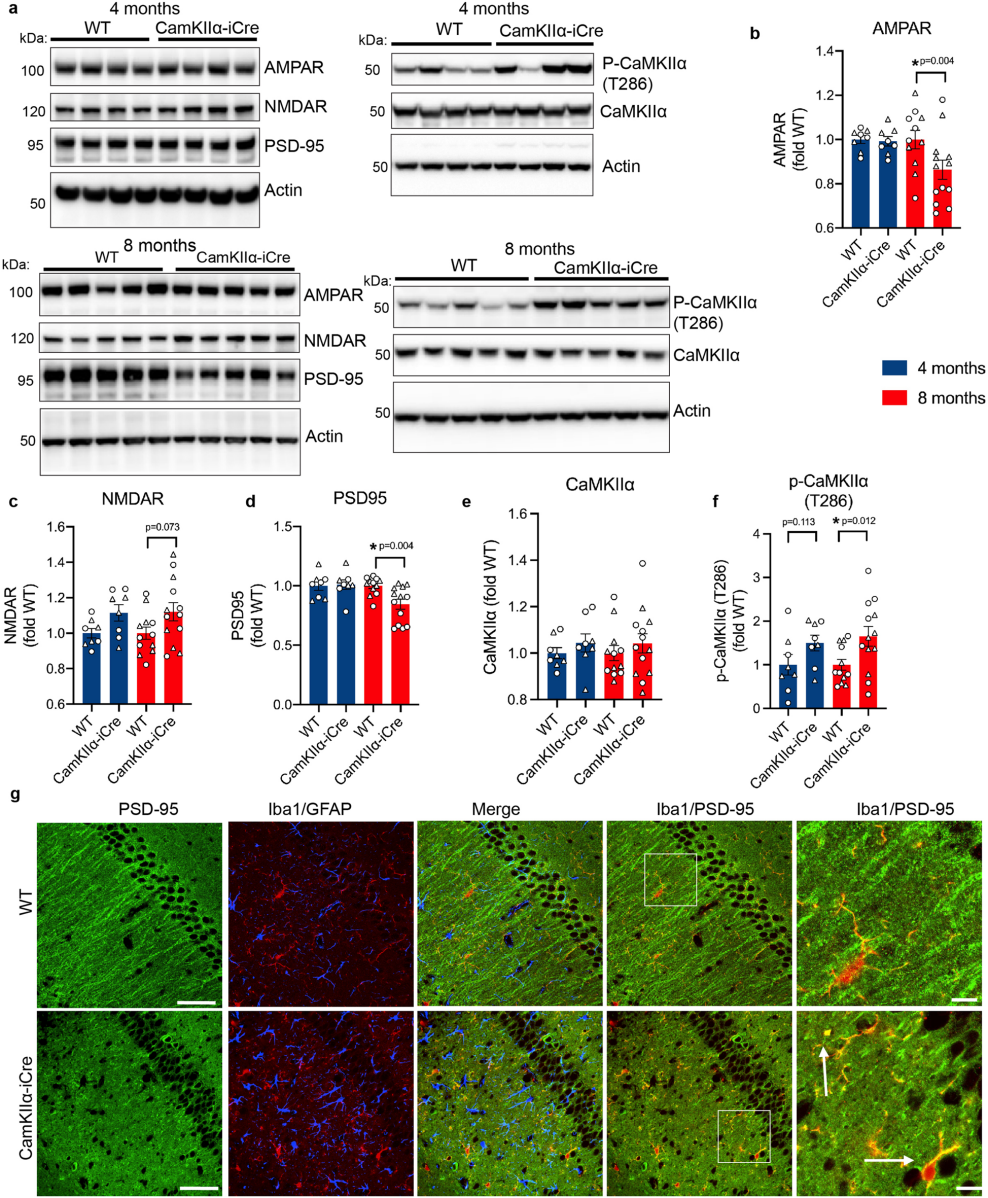
Post-synaptic proteins are altered in the hippocampus of CaMKIIα-iCre mice. **(A)** Immunoblot of hippocampal homogenates from 4-month-old (upper) and 8-month-old (lower) WT and CaMKIIα-iCre mice probed for AMPAR, NMDAR, PSD95, phosphorylated CaMKIIα (Thr286), total CaMKIIα and actin. Original immunoblots of 4-month-old mice are shown in Fig. S4A. Original immunoblots of 8-month-old mice are shown in Fig. S4B. Quantification of AMPAR **(B)**, NMDAR **(C)**, PSD95 **(D)**, total CaMKIIα **(E)** and phosphorylated CaMKIIα (Thr286) **(F)** immunoblots in A normalized to actin and expressed as fold of WT (WT, n = 12; CaMKIIα-iCre, n = 13). **(G)** Immunofluorescence microscopy of representative coronal brain sections showing the CA1 hippocampus from 8-month-old WT and CamKIIα-iCre mice labeled for PSD95 (green), Iba1 (red) and GFAP (blue), scale bars 50 µm. Magnified images show strong co-localization (yellow) between PSD95 (green) and Iba1 (red) depicted by arrows, scale bars 10 µm. Circles = females, triangles = males. Unpaired t-test was performed for all quantifications and values are mean ± SEM.

To investigate this hypothesis, we first tested expression levels of post-synaptic proteins enriched in the SR and SO layers in hippocampal brain homogenates of 4 and 8-month-old CaMKIIα-iCre and WT mice. Ionotropic glutamate receptors AMPAR and NMDAR, postsynaptic density protein 95 (PSD95), CaMKIIα and phosphorylated CaMKIIα (Thr286) were measured by immunoblot analyses (Fig. 3A). No significant differences in PSD95, AMPAR or NMDAR were detected between 4-month-old WT and CaMKIIα-iCre mice (Fig. 3B–D), whereas in 8-month-old CaMKIIα-iCre mice, the expression levels of PSD95 and AMPAR were significantly decreased, while NMDAR was increased (Fig. 3B–D). The total level of CaMKIIα in hippocampal brain homogenates of 4 or 8 month-old CaMKIIα-iCre mice compared to WT mice was unchanged (Fig. 3E), yet the phosphorylation of CaMKIIα at Thr286 was markedly increased in CaMKIIα-iCre mice compared to WT mice (Fig. 3F). To determine whether the changes in synaptic proteins were associated with activated microglia and astrocytes in CaMKIIα-iCre mice, immunofluorescence microscopy was performed for Iba1, GFAP and PSD95 in 8-month-old mice (Fig. 3G). PSD95 staining in dendrites of CA1 neurons projecting into SR hippocampal layers appeared to be reduced and disorganized in CaMKIIα-iCre mice, and an increase in the co-localization of PSD95 and Iba1 was observed, suggesting the possibility of enhanced engulfment of synapses by microglia in CaMKIIα-iCre mice (Fig. 3G).

**Figure 4:**
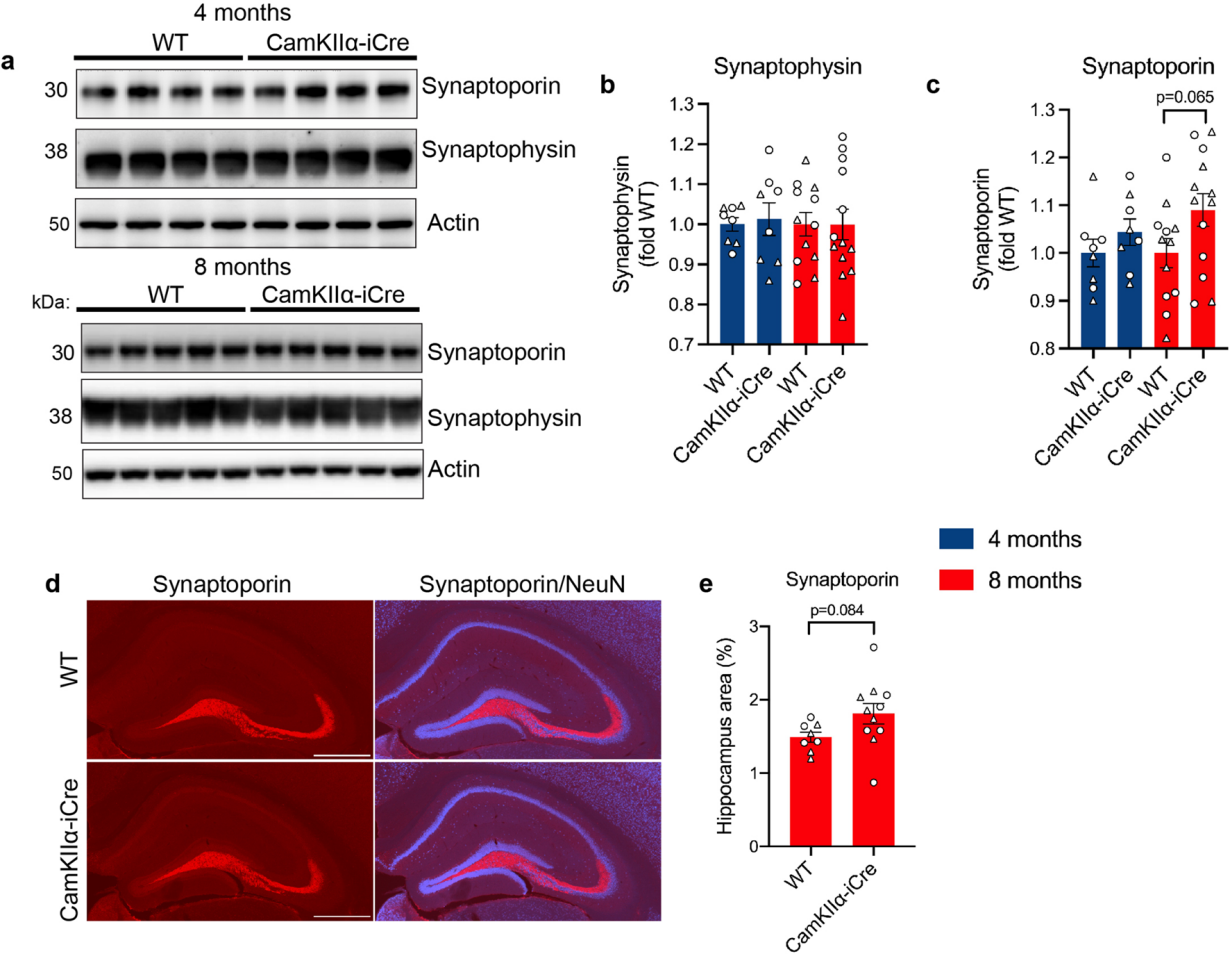
Presynaptic proteins are unchanged in the hippocampus of CaMKIIα-iCre mice. **(A)** Immunoblot of hippocampal homogenates from 4-month-old and 8-month-old male WT and CaMKIIα-iCre mice probed for synaptophysin, synaptoporin and actin. Original immunoblots are shown in Fig. S5A,B. Quantification of synaptophysin **(B)** and synaptoporin **(C)** immunoblots in **(A)** normalized to actin and expressed as fold of WT (WT, n = 12; CaMKIIα-iCre, n = 13). Circles = females, triangles = males. **(D)** Immunofluorescence microscopy of representative coronal brain sections showing the hippocampus from 8-month-old male WT and CaMKIIα-iCre mice labeled for synaptoporin (red) and NeuN (blue). Scale bar, 1000 µm. **(E)** Quantification of synaptoporin immunostaining as a percentage of area covering the hippocampus from WT and CaMKIIα-iCre mice (WT, n = 9; CaMKIIα-iCre, n = 11). Unpaired t-test was performed for all quantifications and values are mean ± SEM.

Presynaptic terminal proteins synaptophysin and synaptoporin are concentrated in the mossy fiber pathway of the hippocampus and were analyzed by immunoblot in 4 and 8-month-old hippocampal homogenates of CaMKIIα-iCre and WT mice (Fig. 4A). Here, no significant differences were observed (Fig. 4B,C), although 8-month-old male CaMKIIα-iCre mice showed a trend towards an increase in synaptoporin levels, and a decrease in synaptophysin levels, compared to WT mice (Fig. S2I–L). Immunofluorescence imaging for synaptoporin and NeuN confirmed the trending increase in synaptoporin in 8-month-old CaMKIIα-iCre mice (Fig. 4D,E). These results suggest synaptic alterations in the hippocampal regions where activated microglia and astrocytes are found to associate with post synaptic regions that express iCre, such as in the SR and SO.

### Hippocampal volume is reduced in aged CaMKIIα-iCre mice

**Figure 5:**
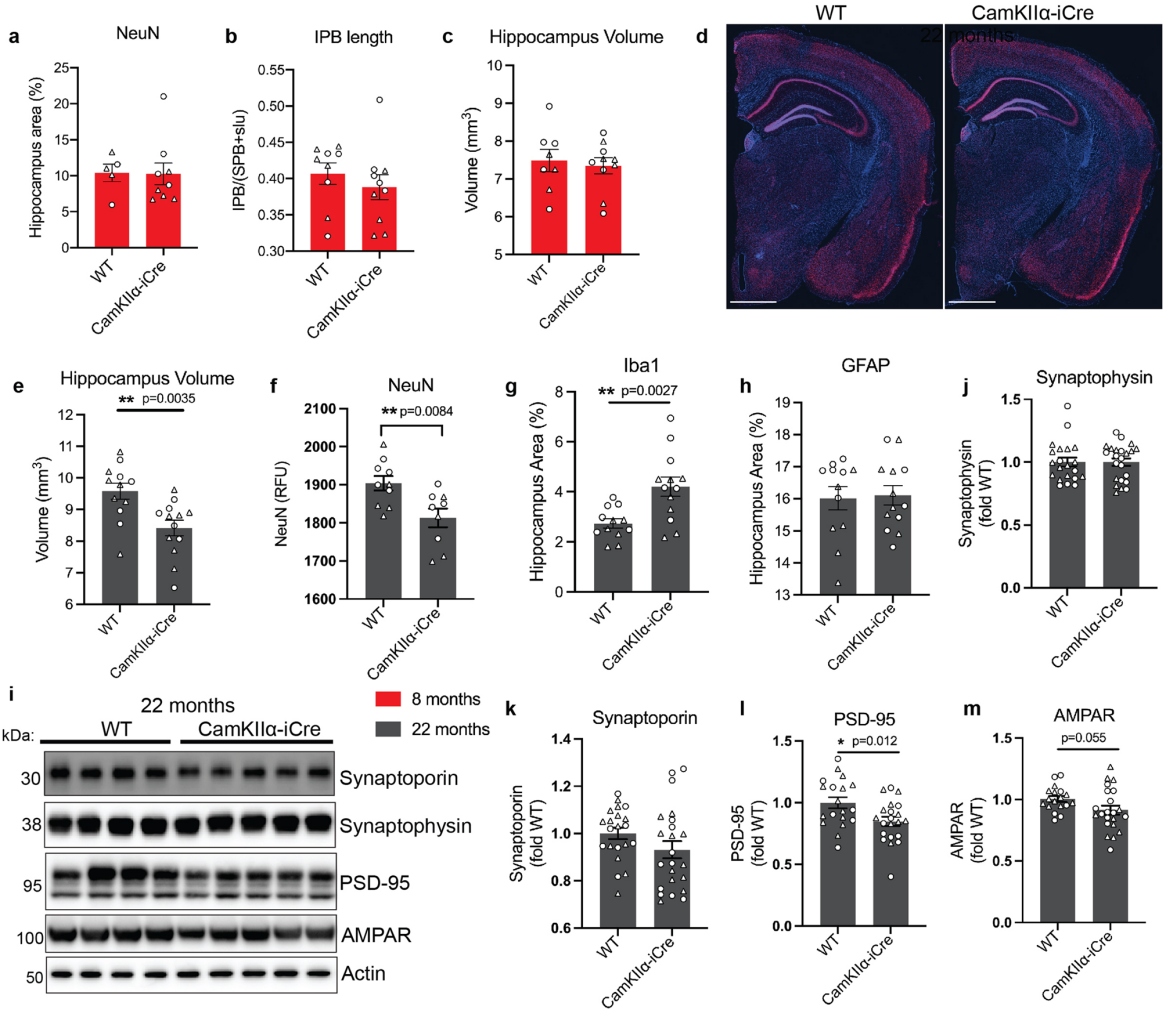
Hippocampal volume is reduced in aged CaMKII-iCre mice. Quantifications of NeuN covered area **(A)** and intrapyramidal bundle (IPB) length **(B)** in the hippocampus of 8-month-old WT and CaMKIIα-iCre mice. Immunofluorescence microscopy of representative coronal brain sections for synaptoporin and NeuN are shown in Fig. 4D. IPB lengths were normalized to the length of the suprapyramidal bundle (SPB) and stratum lucidum (slu) and expressed as a ratio of IPB/SPB + slu. **(C)** Quantification of hippocampus volumes (mm^3^) in 8-month-old WT and CaMKIIα-iCre mice. **(D)** Representative coronal brain sections from female 22-month-old WT and CaMKIIα-iCre mice labeled for NeuN (red) and DAPI (blue). Scale bar, 1000 µm. **(E)** Quantification of hippocampus volumes (mm^3^) in 22-month-old WT and CaMKIIα-iCre mice. **(F)** Quantification of NeuN immunostaining in the hippocampus of 22-month-old WT and CaMKIIα-iCre mice. Quantification of Iba1 **(G)** or GFAP **(H)** percentage of area covering the hippocampus from WT and CaMKIIα-iCre mice (WT, n = 12; CaMKIIα-iCre, n = 13). **(I)** Immunoblot of hippocampal homogenates from 22-month-old WT and CaMKIIα-iCre mice probed for synaptophysin, synaptoporin, PSD-95, AMPAR and actin. Original immunoblots are shown in Fig. S6. Quantification of synaptophysin **(J)**, synaptoporin **(K)**, PSD95 **(L)** and AMPAR **(M)** immunoblots in **(I)** normalized to actin and expressed as fold of WT. (WT, n = 17; CaMKIIα-iCre, n = 20). Circles = females, triangles = males. Unpaired t-test was performed for all quantifications and values are mean ± SEM.

The levels of the post-synaptic proteins in hippocampal homogenates of WT and CaMKIIα-iCre mice were comparable at 4-months, but altered at 8-months, suggesting that synaptic defects in CaMKIIα-iCre mice were caused by the chronic activation of microglia and astrocytes (Fig. 3B–D). To investigate whether hippocampal atrophy followed synaptic defects in CaMKIIα-iCre mice, hippocampus volume was first assessed in 8 and 22-month-old WT and CaMKIIα-iCre mice. The length of the intra pyramidal bundle (IPB) and NeuN immunostaining were also quantified to assess mossy fiber axon organization and neurodegeneration, respectively. IPB length, NeuN covered area and hippocampus volume were unchanged between 8-month-old CaMKIIα-iCre mice and WT mice (Fig. 5A–C), indicating that glial activation and synaptic alterations did not cause hippocampal atrophy in the CaMKIIα-iCre hippocampus at 8-months.

However, when hippocampus volume was measured in CaMKIIα-iCre mice at 22-months, a significant reduction was observed compared to WT mice (Fig. 5D,E). NeuN immunostaining was also significantly reduced in 22-month-old CaMKIIα-iCre mice (Fig. 5F). To determine whether elevated Iba1 and GFAP persisted in 22-month-old CaMKIIα-iCre mice, we measured Iba1 and GFAP covered area in hippocampal brain regions by immunostaining. A significant increase in Iba1 was observed (Fig. 5G), although GFAP levels were similar between WT and CaMKIIα-iCre mice (Fig. 5H). GFAP covered area increased with age in WT mice (13.75% GFAP covered area at 8 months (Fig. 1B) versus 16.01% GFAP covered area at 22 months (Fig. 5H)), whereas GFAP covered area was unchanged with age in CaMKIIα-iCre mice (Figs. 1B, 5H), which may explain the equivalent GFAP levels in WT and CaMKIIα-iCre mice at 22 months. Finally, presynaptic proteins synaptoporin and synaptophysin were unchanged (Fig. 5I–K), while PSD-95 and AMPAR decreased in 22-month-old mice (Fig. 5I,L,M). Altogether, our data suggest that reactive microglia and astrocytes proliferate to the SR and SO hippocampal layers and lead to abnormal expression of postsynaptic proteins, resulting in hippocampal atrophy in aged CaMKIIα-iCre mice.

### Glial activation in hippocampal and cortical brain regions of nestin-Cre mice

To determine whether the increase in activated microglia and astrocytes and synaptic defects observed in the CaMKIIα-iCre mouse line were induced by Cre recombinase itself, and thus common to various Cre mouse strains, we evaluated microglia, astrocytes, and synaptic proteins in the brains of additional Cre-driver mice that express Cre in the hippocampus and cortex. The original CaMKIIα-Cre T29-1 strain^10^ that expresses prokaryotic Cre recombinase in the forebrain at a lower level than in the CaMKIIα-iCre mouse^16^ and nestin-Cre mice^21^ that express high levels of Cre in CNS neuronal precursors were chosen for analysis.

**Figure 6:**
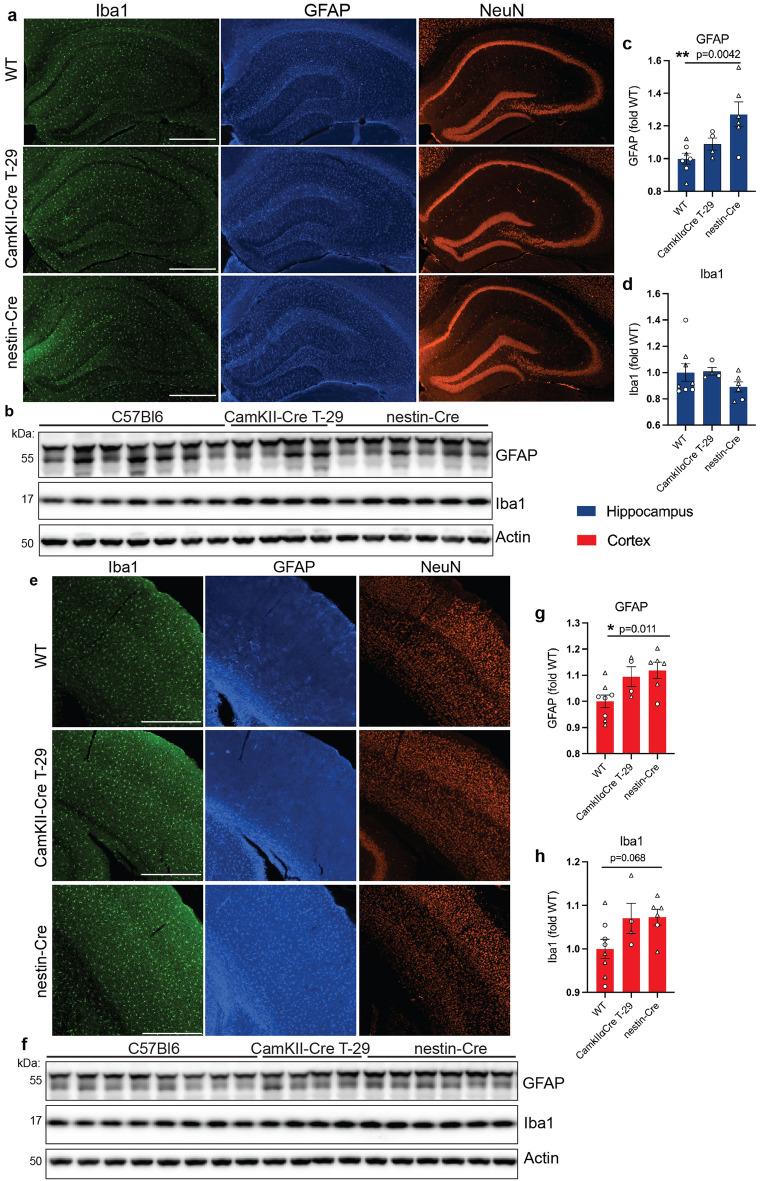
Astrocytosis is increased in hippocampal and cortical brain regions of nestin-Cre mice. **(A)** Immunofluorescence microscopy of representative coronal brain sections showing the hippocampus from 4-month-old wild-type (WT), CaMKIIα-Cre T29-1 and nestin-Cre mice labeled for Iba1 (green), GFAP (blue) and NeuN (red), scale bars 200 µm. **(B)** Immunoblot of hippocampal homogenates from 4-month-old WT, CaMKIIα-Cre T29-1 and nestin-Cre mice probed for GFAP, Iba1 and actin. Original immunoblots are shown in Fig. S7A. Quantification of GFAP **(C)** and Iba1 **(D)** immunoblots in **(B)** normalized to actin and expressed as fold of WT (WT, n = 8; CaMKIIα-Cre T29-1 n = 4; nestin-Cre, n = 6). **(E)** Immunofluorescence microscopy of representative coronal brain sections showing the cortex from 4-month-old WT, CaMKIIα-Cre T29-1 and nestin-Cre mice labeled for Iba1 (green), GFAP (blue) and NeuN (red), scale bars 200 µm. **(F)** Immunoblot of cortical homogenates from 4-month-old WT, CaMKIIα-Cre T29-1 and nestin-Cre mice probed for GFAP, Iba1 and actin. Original immunoblots are shown in Fig. S7B. Quantification of GFAP **(G)** and Iba1 **(H)** immunoblots in **(F)** normalized to actin and expressed as fold of WT (WT, n = 8; CaMKIIα-Cre T29-1, n = 4; nestin-Cre, n = 6). One-way ANOVA followed by Dunnet’s posttest was performed for all quantifications and values are mean ± SEM.

4-month-old CaMKIIα-Cre T29-1 and nestin-Cre mice were first analyzed by immunofluorescence imaging and immunoblotting for Iba1 and GFAP in hippocampal and cortical brain regions. Notably, the distinct proliferation of GFAP and Iba1 to the SR and SO regions of the hippocampus in CaMKIIα-iCre mice (Fig. 1A) was absent in the CaMKIIα-Cre T29-1 strain (Fig. 6A). The localization of Iba1-positive microglia and GFAP-positive astrocytes in hippocampal brain regions also appeared to be similar between nestin-Cre and WT control mice (Fig. 6A). Immunoblot analyses confirmed no differences in the levels of GFAP or Iba1 in hippocampal brain regions of CaMKIIα-Cre T29-1 mice compared to WT mice (Fig. 6B–D). A significant increase in GFAP in the hippocampus was found in nestin-Cre mice (Fig. 6C).

We next analyzed Iba1 and GFAP expression in cortical brain regions of CaMKIIα-Cre T29-1 and nestin-Cre mice. No changes in Iba1 or GFAP distribution or total covered area in CaMKIIα-Cre T29-1 mice compared to WT controls were observed by immunofluorescence microscopy (Fig. 6E). However, GFAP expression was markedly increased in cortical brain regions in nestin-Cre mice compared to WT or CaMKIIα-Cre T29-1 mice (Fig. 6E). There was also a trend towards an increase in Iba1 in cortical brain regions of nestin-Cre mice, compared to WT or CaMKIIα-Cre T29-1 mice (Fig. 6E,H). The elevations of GFAP and Iba1in the cortex of nestin-Cre mice were confirmed by immunoblot analyses (Fig. 6F–H).

### Synaptic protein changes in the hippocampus and cortex of nestin-Cre mice

**Figure 7:**
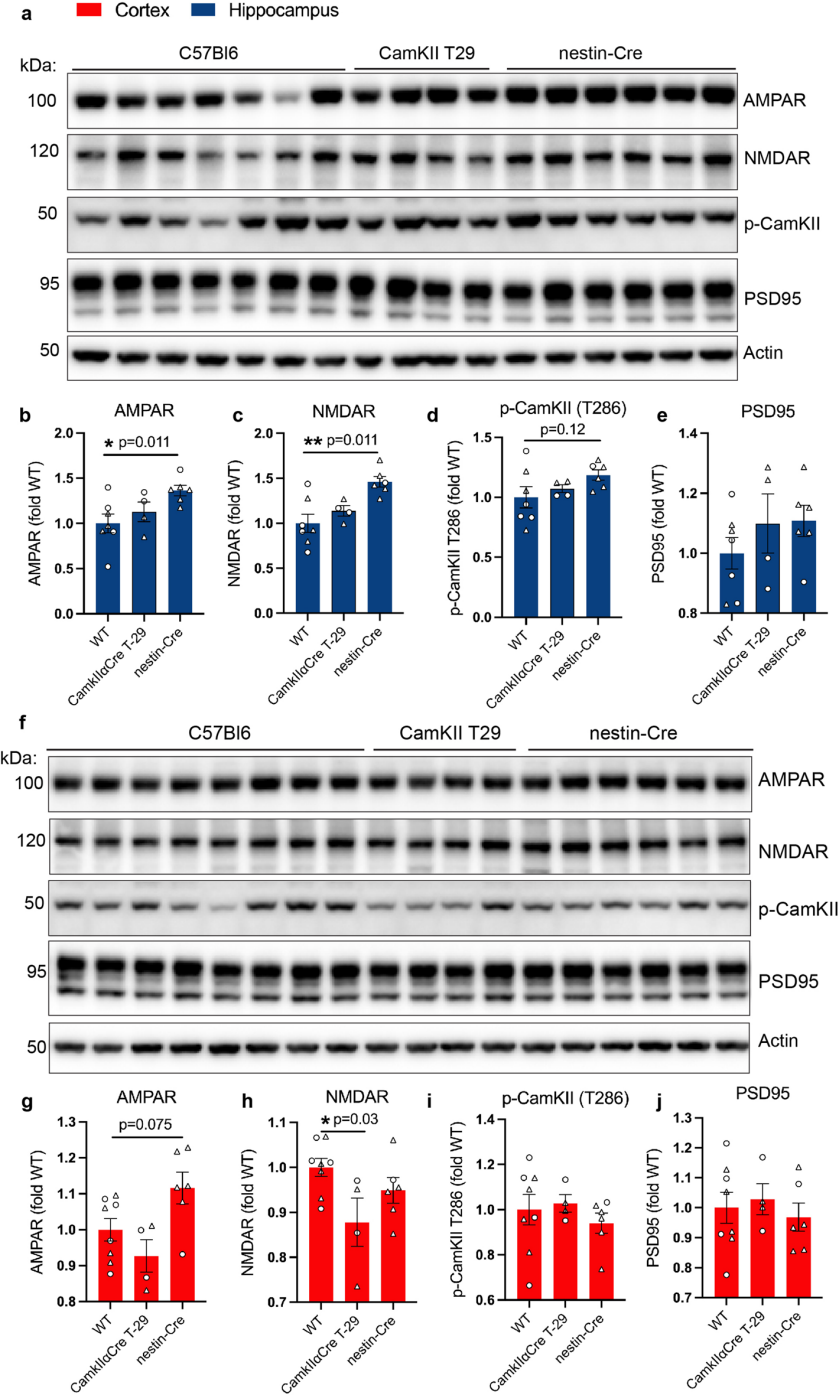
Synaptic proteins are altered in hippocampal brain regions of nestin-Cre mice. **(A)** Immunoblot of hippocampal homogenates from 4-month-old wild-type (WT), CaMKIIα-Cre T29-1 and nestin-Cre mice probed for AMPAR, NMDAR, phospho-CaMKII (T286), PSD95 and actin. Original immunoblots are shown in Fig. S8A. Quantification of AMPAR **(B)**, NMDAR **(C)**, phospho-CaMKII (T286) **(D)** and PSD95 **(E)** immunoblots in **(A)** normalized to actin and expressed as fold of WT (WT, n = 7; CaMKIIα-Cre T29-1, n = 4; nestin-Cre, n = 6). **(F)** Immunoblot of cortical homogenates from 4-month-old WT, CaMKIIα-Cre T29-1 and nestin-Cre mice probed for AMPAR, NMDAR, phospho-CaMKII (T286), PSD-95 and actin. Original immunoblots are shown in Fig. S8B. Quantification of AMPAR **(G)**, NMDAR **(H)**, phospho-CaMKII (T286) **(I)** and PSD95 **(J)** immunoblots in **(F)** normalized to actin and expressed as fold of WT (WT, n = 8; CaMKIIα-Cre T29-1, n = 4; nestin-Cre, n = 6). One-way ANOVA followed by Dunnet’s posttest was performed for all quantifications and values are mean ± SEM.

We found changes in postsynaptic proteins accompanying microgliosis and astrocytosis in the hippocampus in CaMKIIα-iCre mice. Therefore, we next checked the cortex and hippocampus of nestin-Cre and CaMKIIα-Cre T29-1 mice for expression levels of postsynaptic markers. PSD-95, AMPAR, NMDAR and phosphorylated CaMKII were affected in CaMKIIα-iCre mice (Fig. 3) although unaffected in WT or CaMKIIα-Cre T29-1 mice in the hippocampus (Fig. 7A–E), which is consistent with the comparable expression of GFAP and Iba1 in hippocampal brain regions of CaMKIIα-Cre T29-1 mice and WT mice (Fig. 6). By contrast, Nestin-Cre mice presented with dramatic increases in AMPAR (Fig. 7B), NMDAR (Fig. 7C), and trends toward an increase phosphorylated CaMKII (Fig. 7D) and PSD95 (Fig. 7E). In cortical brain regions, immunoblot analyses showed trends toward a decline in AMPAR and NMDAR expression in CaMKIIα-Cre T29-1 mice compared to WT mice (Fig. 7F–H), while PSD95 and phosphorylated CaMKII levels remained stable (Fig. 7F,I,J). No significant differences in NMDAR, phosphorylated CaMKII and PSD95 were observed in cortical brain regions of nestin-Cre mice, while a trend toward an increase in AMPAR was observed. These results suggest the possibilities that activation of astrocytes had a more robust impact on hippocampal synaptic proteins compared to cortical proteins. It is also possible that changes in synaptic proteins may occur in a manner independent of glial activation in the nestin-Cre mouse.

## Discussion

The results in our study establish additional undesirable glial and synaptic changes in mice expressing Cre under control of the CNS promoters CaMKIIα and nestin. In CaMKIIα-iCre mice increased microglial activation as well as heightened astrocytic reactivity occurred in pyramidal, SR and SO hippocampal regions of male and female mice, yet only presented in the cortex of female CaMKIIα-iCre mice. The highly activated state of microglia and astrocytes appeared to reside within layers of the hippocampus that comprise significant populations of interneurons that control excitatory synaptic activity of neural circuits^33^. We speculate the homeostatic state within neural circuits of the hippocampus is disrupted within CaMKIIα-iCre mouse brains. While no changes in presynaptic protein expression of synaptophysin and synaptoporin were observed, alterations in post-synaptic proteins NMDAR, phosphorylated CaMKIIα, PSD95 and AMPAR were likely induced by the long-term state of neuroinflammation characterized by active and reactive microglia and astrocytes. We hypothesize that this chronic inflammation in the hippocampus eventually led to hippocampal atrophy in aged CaMKIIα-iCre mice.

We sought to identify whether neuroinflammation and synaptic changes were limited to CaMKIIα-iCre mice by evaluating the brains of additional common Cre-driver mouse models. We assessed the original CaMKIIα-Cre T29-1 strain^10^ and the nestin-Cre mouse model^21^. We chose to assess these two strains because they are widely used and express the prokaryotic version of Cre, thus allowing us to investigate whether neuroinflammation and synaptic changes in the CaMKIIα-iCre mice were caused by features unique to iCre. The nestin-Cre model expresses Cre within neural progenitors, while the CaMKIIα-Cre T29-1 strain expresses Cre in forebrain excitatory neurons. Unexpectedly, nestin-Cre mice showed enhanced astrocytic infiltration into cortical and hippocampal brain regions compared to WT controls and we found significant changes in hippocampal AMPARs and NMDARs. In contrast, the levels of GFAP and Iba1 were similar in hippocampal and cortical brain regions of CaMKIIα-Cre T29-1 and WT mice. The mechanism underlying glial activation in the nestin-Cre and CaMKIIα-iCre mice is unknown but may possibly cause downstream synaptic changes and neurodegeneration. Together, our results have broad implications on the interpretation of results derived from studies using CNS Cre-driver mice for gene manipulation, especially those focused on glial or synaptic processes in nestin-Cre and CaMKIIα-iCre mice.

Interestingly, we noted the location of microglia and astrocytes surrounding the synapses of neural circuits in the hippocampus of CaMKIIα-iCre mice, as well as changes in synaptic proteins that are involved in glutamatergic excitatory neurotransmission. Considering the co-localization of postsynaptic protein PSD95 within microglial soma, we hypothesize that activated microglia alter excitatory synapses in the hippocampus, ultimately impeding signal transduction within synapses. This process is mediated by NMDARs that control calcium influx into the post synapse and regulate the postsynaptic targeting of AMPARs. CaMKIIα is regulated by calcium levels and is highly enriched in postsynaptic densities of neuronal synapses and is a key protein involved in regulating synaptic physiology of the hippocampus^34^. The activity of CaMKIIα is controlled by its autophosphorylation at Thr286 in response to prolonged elevation of intracellular calcium levels, which is critical for LTP and memory in mice^35,36^. We observed concomitant changes in AMPARs and NMDARs, and an increase in CaMKIIα phosphorylation in CaMKIIα-iCre mice, so it is possible that constitutive activity of CaMKIIα due to increased autophosphorylation could be a critical event in driving the association of microglia and astrocytes with postsynaptic regions. Signal transduction within synapses of the hippocampus is critical for LTP and LTD and is required for synaptic plasticity and memory storage in the brain, so it is possible that these changes led to hippocampal-dependent behavioral and electrophysiological alterations in CaMKIIα-iCre mice.

We observed neuroinflammatory and neurodegenerative changes in CaMKIIα-iCre mice that express iCre and not in CaMKIIα-Cre T29-1 mice that express prokaryotic Cre. iCre expresses at a higher level than prokaryotic Cre when equal amounts of expression vectors are used^16^, and in nestin-Cre mice that express Cre in all neural progenitors, GFAP and Iba1 increased in Cre expressing brain areas. These observations support the hypothesis that high Cre expression causes neuroinflammation in CaMKIIα-iCre mice and in nestin-Cre mice. It is possible that neuroinflammation may be caused by apoptosis of Cre-expressing neurons, or by altered synaptic activity of Cre neurons leading to the activation of synaptic pruning by microglia. Cre has been previously known to have pathological effects; Cre decreases cell proliferation and increases cell apoptosis in vitro in cultured cell lines^24,37,38^ and Cre-mediated damage to nestin-expressing neural progenitors results in microencephaly and hydrocephalus in vivo in nestin-Cre mice^20,39^. The timeline of pathology in CaMKIIα-iCre mice indicates that activated microglia and astrocytes lead to synaptic alterations resulting in synapse loss and brain atrophy. The correlation between Cre levels, neuroinflammation and neurodegeneration is not fully understood and should be investigated in further studies.

If Cre expression level led to neuroinflammation in nestin-Cre and CaMKIIα-iCre mice, it is possible that our findings could extend to other Cre-driver mice that express high levels of Cre driven by neuronal promoters. Furthermore, Cre expression driven by glial cell promoters may directly affect the activity of microglia and astrocytes. A limitation of this study is that we did not manipulate Cre expression in nestin-Cre and CaMKIIα-iCre mice, as this would likely clarify whether Cre-expression caused the phenotypes observed in our study. Future studies to address this could be performed by pharmacologically inhibiting Cre, comparing homozygous and heterozygous genotypes, or using Cre inducible tamoxifen-treated mice. Moreover, we evaluated CaMKIIα-iCre mice 4, 8 and 22-months-old, while nestin-Cre and CaMKIIα-Cre T29-1 mice were only examined at 4-months-old. Neuroinflammation in CaMKIIα-iCre mice occurred at 4-months-old, although synaptic changes and hippocampal atrophy did not present until mice were 8 months old and 22-months-old, respectively. It is possible that aging may increase the level of Cre and lead to the more severe phenotypes observed in 8 and 22-month-old CaMKIIα-iCre mice, or that chronic neuroinflammation with age leads to downstream synaptic changes and neuronal loss. Future studies utilizing various strains of heterozygous and homozygous CNS Cre-driver mice should be performed in young and old mice to address these questions.

Other indirect mechanisms that are specific to nestin-Cre and CaMKIIα-iCre mice, and not associated directly with Cre expression, may possibly explain our findings. For instance, Cre transgenic mouse models are typically created by pronuclear microinjection of Cre transgenes, wherein the genomic insertion site of the Cre transgene is random and could interfere with endogenous gene expression or lead to multiple copies of Cre within the same chromosome^40^. In addition, Cre expression has caused unexpected results in Cre-driver mice by performing illegitimate recombination of genes through pseudo lox-P sites^41^. In a recent study examining the overexpression of synaptotagmin-2 in CaMKIIα-iCre, the BAC transgene insertion was hypothesized to disrupt the suppression machinery of the syt2 locus, leading to ectopic overexpression^25^. Therefore, it is conceivable that the expression of endogenous genes was disrupted, either by illegitimate recombination or the random insertion of the Cre transgene. Disruption of loci involved in glial or synaptic function by Cre transgene insertion could potentially lead to the neuroinflammation seen in nestin-Cre and CaMKIIα-iCre mice.

The methods used to create the nestin-Cre and CaMKIIα-iCre mouse models could have also caused the unexpected neuroinflammatory and synaptic alterations in these mice. One key difference between the CaMKIIα-iCre mouse model and other CaMKIIα Cre mice, including the CaMKIIα-Cre T29-1 strain, is the construction and expression of the Cre transgene in a large 170 kb BAC as opposed to use of a traditional small promoter-based strategy. The BAC is superior in that it supports desired endogenous gene expression by containing all required regulatory elements^11^, though the unintentional overexpression of additional genes located in the arms of the BAC may warrant unwanted phenotypes in CaMKIIα-iCre mice. The CaMKIIα-iCre BAC contains genomic sequence 50 kb upstream and 100 kb downstream of the CaMKIIα gene^11^ and based on the chromosomal regions surrounding the CaMKIIα locus (https://www.ncbi.nlm.nih.gov/gene/12322), the genes *SLC6A7*, *ARSI* and *CDX1* were likely to be incorporated into the BAC and are overexpressed in CaMKIIα-iCre mice^25^. Although these are genes not directly involved with astrocytes or microglia, our results may be linked to the overexpression of *SLC6A7* which is a member of the gamma-aminobutyric acid neurotransmitter gene family and encodes a high-affinity mammalian brain L-proline transporter protein^42^. Although reports regarding the function of SLC6A7 are limited, it is known to be expressed in forebrain synaptic terminals involved in glutamatergic pathways^43^, which could lead to inappropriate synaptic activity and activated glia-mediated synaptic pruning.

The nestin-Cre mouse has long been established as having serious adverse physiological problems, the most prominent being microencephaly and hydrocephalus, which have been attributed to Cre toxicity in neural progenitors^20,23,39^. Other physiological issues in nestin-Cre mice include behavioral abnormalities, hypopituitarism, decreased levels of growth hormone and reduced body weight^44,45^. These artifacts may be associated with endogenous gene disruption by random integration of the Cre transgene^23^. However, the construct used to generate the nestin-Cre mouse model has also been implicated^46^. The transgene carries the human growth hormone (hGH) minigene, as it was identified that the sequences incorporated were critical to efficiently express the Cre transgene under the nestin promoter^47^. The expression of hGH on the nestin-Cre transgene results in aberrant hGH expression in the hypothalamus, and subsequently may cause hypopituitarism, metabolic and behavioral phenotypes in nestin-Cre mice^46^. Importantly, hGH is known to regulate glial and neuronal cell differentiation in the CNS^48^. Glial cells express the hGH receptor (GHR) and in GHR deficient mice, astrocytes are smaller and fewer in number^49^. It is unknown whether hGH is overexpressed in the cortex or hippocampus of nestin-Cre mice, but it is possible that overexpression of hGH in nestin-Cre mice could have caused enhanced astrocytic activity. Therefore, while we hypothesize that high Cre levels induce neuroinflammation, additional genes contained on the transgenes in the nestin-Cre mouse and in the CaMKIIα-iCre mouse could be responsible for the results observed in our study.

The use of tissue and cell specific promoters to express Cre in transgenic mice has become a staple for studying the genetics of diseases in translational research. The knowledge gathered thus far would not have been conceivable without the availability of tools such as Cre recombinase to manipulate genetic expression in mice. As the use of Cre-driver mice continues, the possibility of arising phenotypes because of inappropriate genetic recombination, Cre toxicity, endogenous gene disruption and germline recombination must be taken into consideration. We identify and report inflammation and synaptic changes present within two widely used Cre models: nestin-Cre and CaMKIIα-iCre mice. The activation of microglia and astrocytes led to further detrimental effects on synapses and neurodegeneration in CaMKIIα-iCre mice. Although we did not analyze nestin-Cre brains at advanced ages for neurodegeneration, a similar cascade of events would likely follow enhanced glial activity in these mice. The CaMKIIα-Cre T29-1 strain did not exhibit any differences compared to WT mice in our study and may be a superior model for genetic manipulation in excitatory forebrain neurons than CaMKIIα-iCre mice, although a lower level of Cre expression may lead to incomplete gene knockout in these mice. Our study is limited by a low number CaMKIIα-Cre T29-1 mice and we only analyzed two additional Cre driver mouse models. Based on our findings, future analyses should be performed to determine whether neuroinflammation is common across Cre-driver mice expressing high levels of Cre in the CNS.

Our results demonstrate aberrant neuroinflammation in two widely used Cre-drivers and have broad implications on studies employing Cre-drivers to investigate genes involved in disorders in which the immune system is implicated, such as Alzheimer’s disease. Moreover, the alterations in PSDs, NMDARs and AMPARs that are fundamental to processes involving synaptic plasticity, learning and memory, indicate dysfunction at synapses and alterations in these processes in nestin-Cre and CaMKIIα-iCre mice. The results presented in this study raise uncertainties to other Cre expressing mouse lines, as it is likely that other CNS phenotypes could exist. For the past decade, pathological reports of Cre expression have called for the use of Cre-driver lines as controls, yet these mice are typically not included when designing breeding strategies. To clearly interpret results, future Cre-loxP studies must employ appropriate Cre expressing controls to account for any confounding phenotypes that could present because of transgenic Cre recombinase expression. Furthermore, a comprehensive characterization of any abnormal results in Cre-driver mice must be documented, and studies should include all breeding strategies to obtain genetically modified mice.

## Supplementary Information

Supplementary Figures.

## Data Availability

The datasets used and/or analysed during the current study available from the corresponding author on reasonable request.

## References

[1] Gu H, Marth JD, Orban PC, Mossmann H, Rajewsky K (1994). Deletion of a DNA polymerase beta gene segment in T cells using cell type-specific gene targeting. Science.

[2] Sternberg N, Hamilton D (1981). Bacteriophage P1 site-specific recombination. I. Recombination between loxP sites. J. Mol. Biol..

[3] Abremski K, Hoess R, Sternberg N (1983). Studies on the properties of P1 site-specific recombination: Evidence for topologically unlinked products following recombination. Cell.

[4] Witten IB (2011). Recombinase-driver rat lines: Tools, techniques, and optogenetic application to dopamine-mediated reinforcement. Neuron.

[5] Gavériaux-Ruff C, Kieffer BL (2007). Conditional gene targeting in the mouse nervous system: Insights into brain function and diseases. Pharmacol. Ther..

[6] Daigle TL (2018). A suite of transgenic driver and reporter mouse lines with enhanced brain-cell-type targeting and functionality. Cell.

[7] Taniguchi H (2011). A resource of Cre driver lines for genetic targeting of GABAergic neurons in cerebral cortex. Neuron.

[8] Harris JA (2014). Anatomical characterization of Cre driver mice for neural circuit mapping and manipulation. Front. Neural Circuits.

[9] Bult CJ (2019). Mouse genome database (MGD) 2019. Nucleic Acids Res..

[10] Tsien JZ (1996). Subregion- and cell type-restricted gene knockout in mouse brain. Cell.

[11] Casanova E (2001). A CamKIIalpha iCre BAC allows brain-specific gene inactivation. Genesis.

[12] Burgin KE (1990). In situ hybridization histochemistry of Ca2+/calmodulin-dependent protein kinase in developing rat brain. J. Neurosci..

[13] Hanson PI, Schulman H (1992). Neuronal Ca2+/calmodulin-dependent protein kinases. Annu. Rev. Biochem..

[14] Wang X, Zhang C, Szábo G, Sun QQ (2013). Distribution of CaMKIIα expression in the brain in vivo, studied by CaMKIIα-GFP mice. Brain Res..

[15] Zalcman G, Federman N, Romano A (2018). CaMKII isoforms in learning and memory: Localization and function. Front. Mol. Neurosci..

[16] Shimshek DR (2002). Codon-improved Cre recombinase (iCre) expression in the mouse. Genesis.

[17] Song AJ, Palmiter RD (2018). Detecting and avoiding problems when using the cre-lox system. Trends Genet..

[18] Becher B, Waisman A, Lu LF (2018). Conditional gene-targeting in mice: Problems and solutions. Immunity.

[19] Luo L (2020). Optimizing nervous system-specific gene targeting with Cre driver lines: Prevalence of germline recombination and influencing factors. Neuron.

[20] Forni PE (2006). High levels of Cre expression in neuronal progenitors cause defects in brain development leading to microencephaly and hydrocephaly. J. Neurosci..

[21] Tronche F (1999). Disruption of the glucocorticoid receptor gene in the nervous system results in reduced anxiety. Nat. Genet..

[22] Briancon N, McNay DE, Maratos-Flier E, Flier JS (2010). Combined neural inactivation of suppressor of cytokine signaling-3 and protein-tyrosine phosphatase-1B reveals additive, synergistic, and factor-specific roles in the regulation of body energy balance. Diabetes.

[23] Harno E, Cottrell EC, White A (2013). Metabolic pitfalls of CNS Cre-based technology. Cell Metab..

[24] Naiche LA, Papaioannou VE (2007). Cre activity causes widespread apoptosis and lethal anemia during embryonic development. Genesis.

[25] Matsuura K, Mohamed HMA, Youssef MMM, Yoshida Y, Yamamoto T (2022). Synaptotagmin 2 is ectopically overexpressed in excitatory presynapses of a widely used CaMKΙΙα-Cre mouse line. iScience.

[26] Jurga AM, Paleczna M, Kuter KZ (2020). Overview of general and discriminating markers of differential microglia phenotypes. Front. Cell Neurosci..

[27] Liddelow SA (2017). Neurotoxic reactive astrocytes are induced by activated microglia. Nature.

[28] Han J, Fan Y, Zhou K, Blomgren K, Harris RA (2021). Uncovering sex differences of rodent microglia. J. Neuroinflamm..

[29] Pfau DR, Hobbs NJ, Breedlove SM, Jordan CL (2016). Sex and laterality differences in medial amygdala neurons and astrocytes of adult mice. J. Comp. Neurol..

[30] Johnson RT, Breedlove SM, Jordan CL (2008). Sex differences and laterality in astrocyte number and complexity in the adult rat medial amygdala. J. Comp. Neurol..

[31] Hong S (2016). Complement and microglia mediate early synapse loss in Alzheimer mouse models. Science.

[32] Stevens B (2007). The classical complement cascade mediates CNS synapse elimination. Cell.

[33] Savić N, Pedarzani P, Sciancalepore M (2001). Medium afterhyperpolarization and firing pattern modulation in interneurons of stratum radiatum in the CA3 hippocampal region. J. Neurophysiol..

[34] Lisman J, Schulman H, Cline H (2002). The molecular basis of CaMKII function in synaptic and behavioural memory. Nat. Rev. Neurosci..

[35] Sanhueza M (2011). Role of the CaMKII/NMDA receptor complex in the maintenance of synaptic strength. J. Neurosci..

[36] Skelding KA, Rostas JA (2009). Regulation of CaMKII in vivo: The importance of targeting and the intracellular microenvironment. Neurochem. Res..

[37] Pfeifer A, Brandon EP, Kootstra N, Gage FH, Verma IM (2001). Delivery of the Cre recombinase by a self-deleting lentiviral vector: Efficient gene targeting in vivo. Proc. Natl. Acad. Sci. U.S.A..

[38] Loonstra A (2001). Growth inhibition and DNA damage induced by Cre recombinase in mammalian cells. Proc. Natl. Acad. Sci. U.S.A..

[39] Qiu L, Rivera-Pérez JA, Xu Z (2011). A non-specific effect associated with conditional transgene expression based on Cre-loxP strategy in mice. PLoS ONE.

[40] Lee JY (2006). RIP-Cre revisited, evidence for impairments of pancreatic beta-cell function. J. Biol. Chem..

[41] Schmidt EE, Taylor DS, Prigge JR, Barnett S, Capecchi MR (2000). Illegitimate Cre-dependent chromosome rearrangements in transgenic mouse spermatids. Proc. Natl. Acad. Sci. U.S.A..

[42] Kim JH (2010). A new association between polymorphisms of the SLC6A7 gene in the chromosome 5q31-32 region and asthma. J. Hum. Genet..

[43] Renick SE (1999). The mammalian brain high-affinity L-proline transporter is enriched preferentially in synaptic vesicles in a subpopulation of excitatory nerve terminals in rat forebrain. J. Neurosci..

[44] Galichet C, Lovell-Badge R, Rizzoti K (2010). Nestin-Cre mice are affected by hypopituitarism, which is not due to significant activity of the transgene in the pituitary gland. PLoS ONE.

[45] Giusti SA (2014). Behavioral phenotyping of Nestin-Cre mice: Implications for genetic mouse models of psychiatric disorders. J. Psychiatr. Res..

[46] Declercq J (2015). Metabolic and behavioural phenotypes in nestin-Cre mice are caused by hypothalamic expression of human growth hormone. PLoS ONE.

[47] Orban PC, Chui D, Marth JD (1992). Tissue- and site-specific DNA recombination in transgenic mice. Proc. Natl. Acad. Sci. U.S.A..

[48] Donahue CP, Kosik KS, Shors TJ (2006). Growth hormone is produced within the hippocampus where it responds to age, sex, and stress. Proc. Natl. Acad. Sci. U.S.A..

[49] List EO (2011). Endocrine parameters and phenotypes of the growth hormone receptor gene disrupted (GHR−/−) mouse. Endocr. Rev..

