# Supplementary material for: Aberrant glial activation and synaptic defects in CaMKIIα-iCre and nestin-Cre transgenic mouse models
Source: Sci Rep. 2022 Dec 21;12:22099. doi: 10.1038/s41598-022-26671-4 (PMC9772212; doi:10.1038/s41598-022-26671-4)
Supplement: Supplementary file 1 — Supplementary Figures. [file 41598_2022_26671_MOESM1_ESM.docx]

Aberrant glial activation and synaptic defects in CaMKIIα-iCre and nestin-Cre transgenic mouse models

Alia O. Alia^1^, Sohee Jeon^1^, Jelena Popovic^1^, Miranda A. Salvo^1^ Katherine R. Sadleir^1^, Robert Vassar^1,2^ and Leah K. Cuddy^1^

^1^The Ken and Ruth Davee Department of Neurology, Northwestern University Feinberg School of Medicine, Chicago IL 60611. ^2^Mesulam Center for Cognitive Neurology and Alzheimer's Disease, Northwestern University Feinberg School of Medicine, Chicago IL 60611.


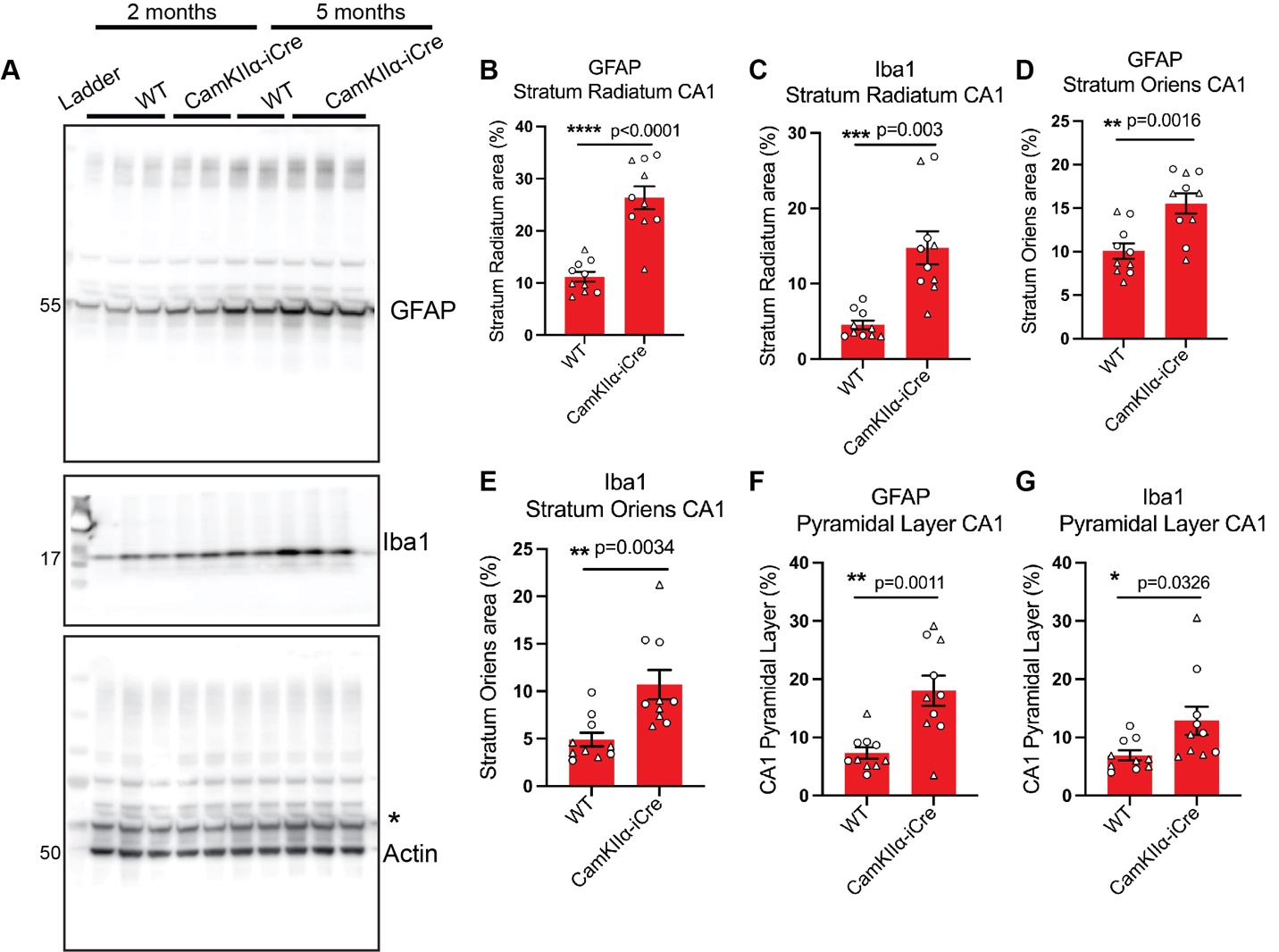


**Supplementary Figure S1.** **A)** Immunoblot of hippocampal homogenates from an additional cohort of 2 and 5-month-old WT and CamKIIα-iCre mice probed for GFAP, Iba1 and Actin. *Denotes remaining residual GFAP signal. GFAP covered area was measured in 8-month-old WT and CamKIIα-iCre in the hippocampal CA1 region in the stratum radiatum **(B)**, stratum oriens **(D)** and pyramidal cell layer **(F)** shown in Fig. 1A.. Iba1 covered area was measured in 8-month-old WT and CamKIIα-iCre in the hippocampal CA1 region in the stratum radiatum **(C)**, stratum oriens **(E)** and pyramidal cell layer **(G)**.


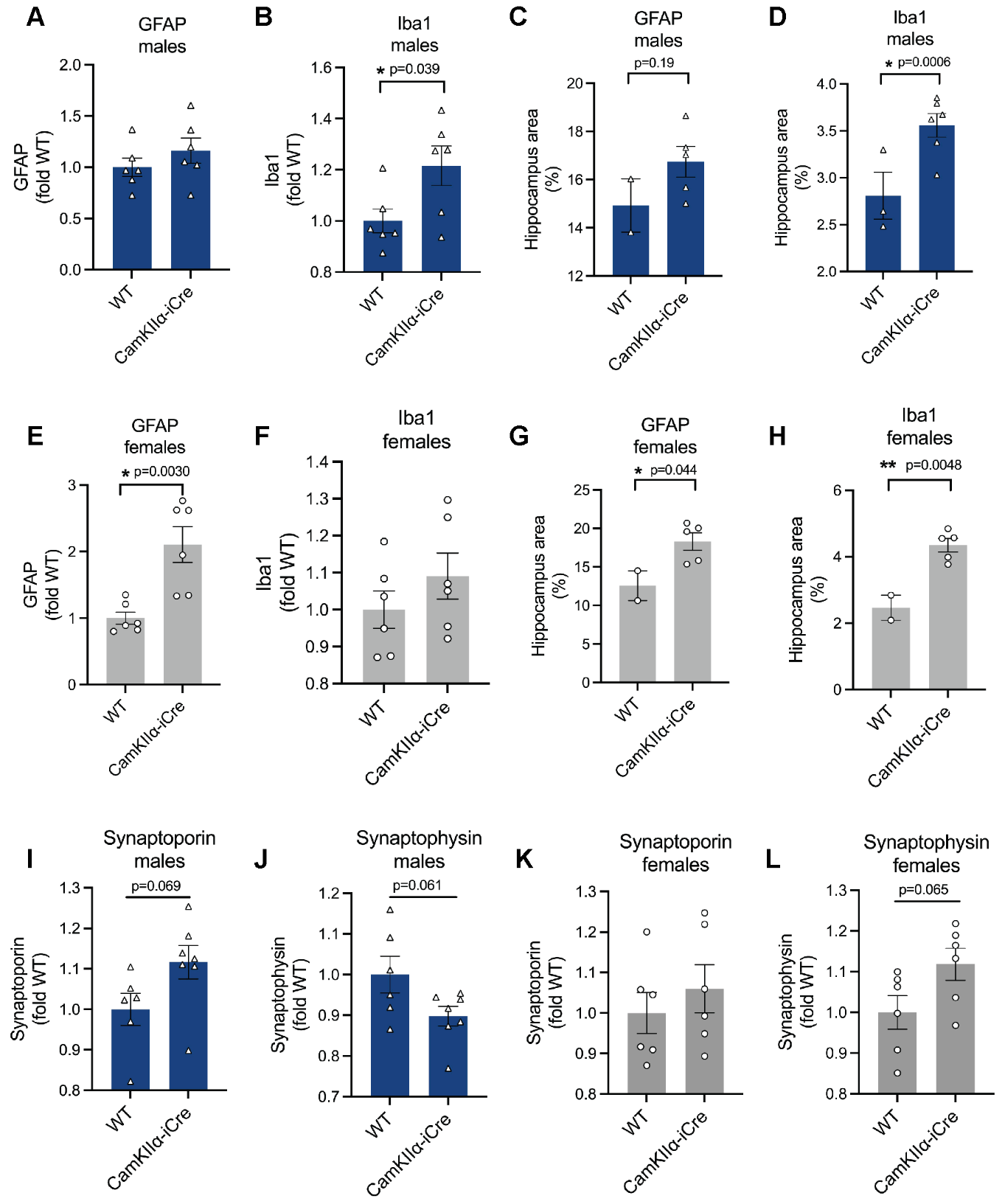


**Supplementary Figure S2.** Quantification of Iba1 and GFAP in the hippocampus from WT and CaMKIIα-iCre mice **(Shown in Fig. 1D, E, H, I)** individually analyzed for males **(A-D)** and females **(E-H).** Quantification of synaptoporin and synaptophysin in the hippocampus from WT and CaMKIIα-iCre mice **(Shown in Fig. 4B, C)** individually analyzed for males **(I, J)** and females **(K, L).**

**
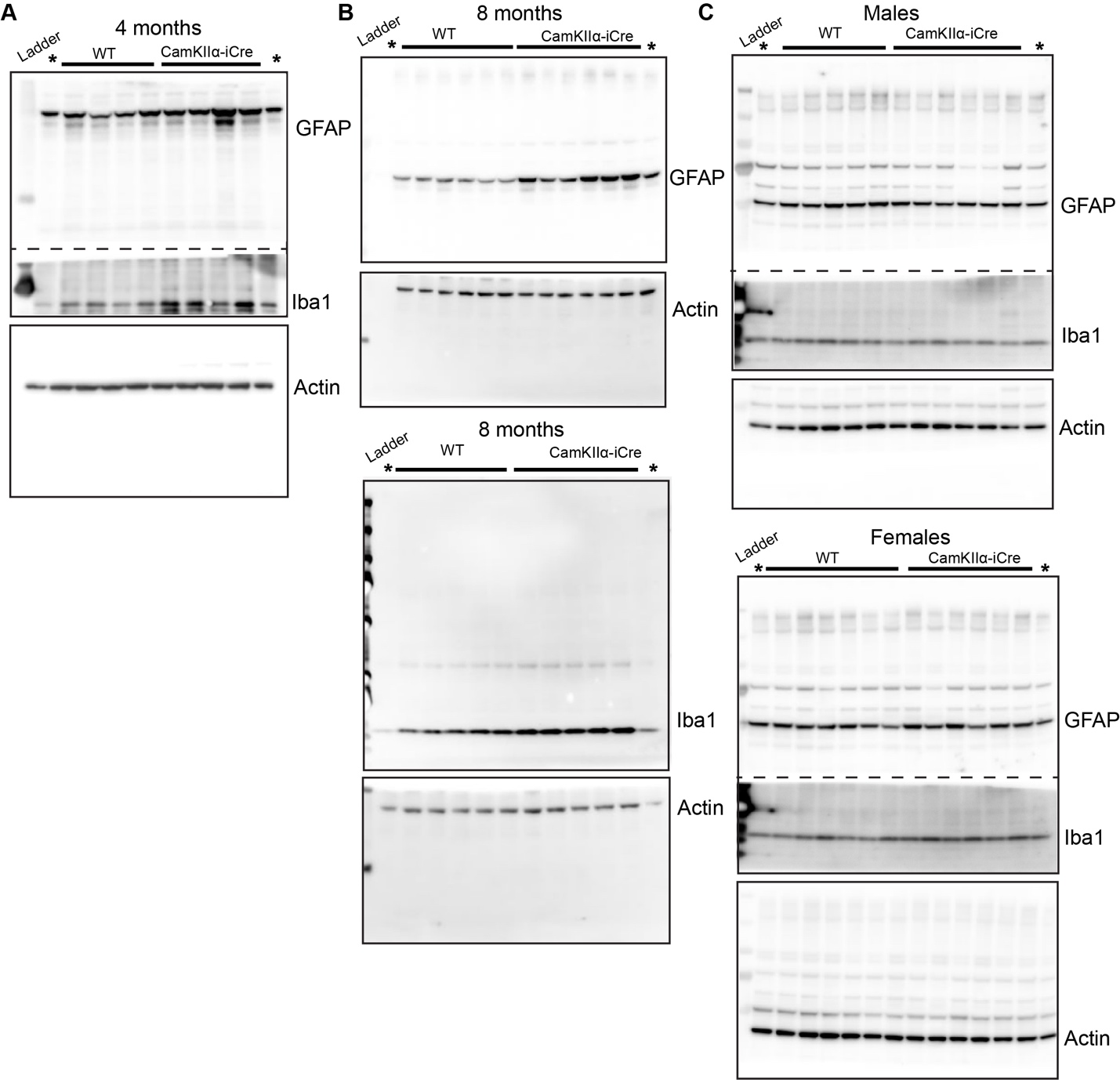
**

**Supplementary Figure S3**. **A)** Original immunoblots of Fig. 1F. **B)** Original blots of Fig. 1G. **C)** Original blots of Fig. 2I. Dashed line indicates blots that were cut and probed with different antibodies. *Denotes lanes shown for clarification of blot edges that were not included in analyses.


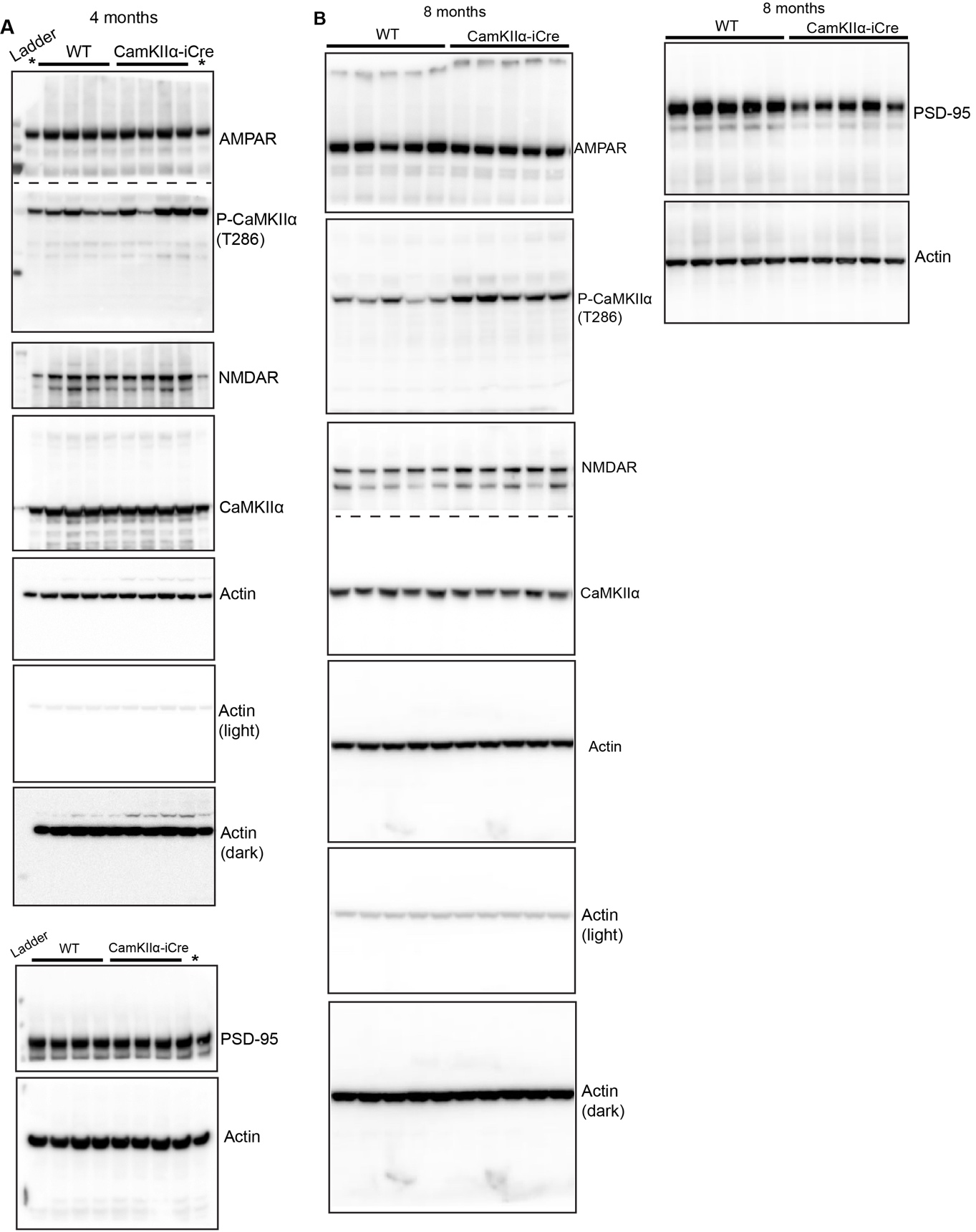


**Supplementary Figure S4**. **A)** Original immunoblots of 4-month-old mice shown in Fig. 3A. **B)** Original immunoblots of 8-month-old mice shown in Fig. 3A. Dashed line indicates blots that were cut and probed with different antibodies. *Denotes lanes shown for clarification of blot edges that were not included in analyses.


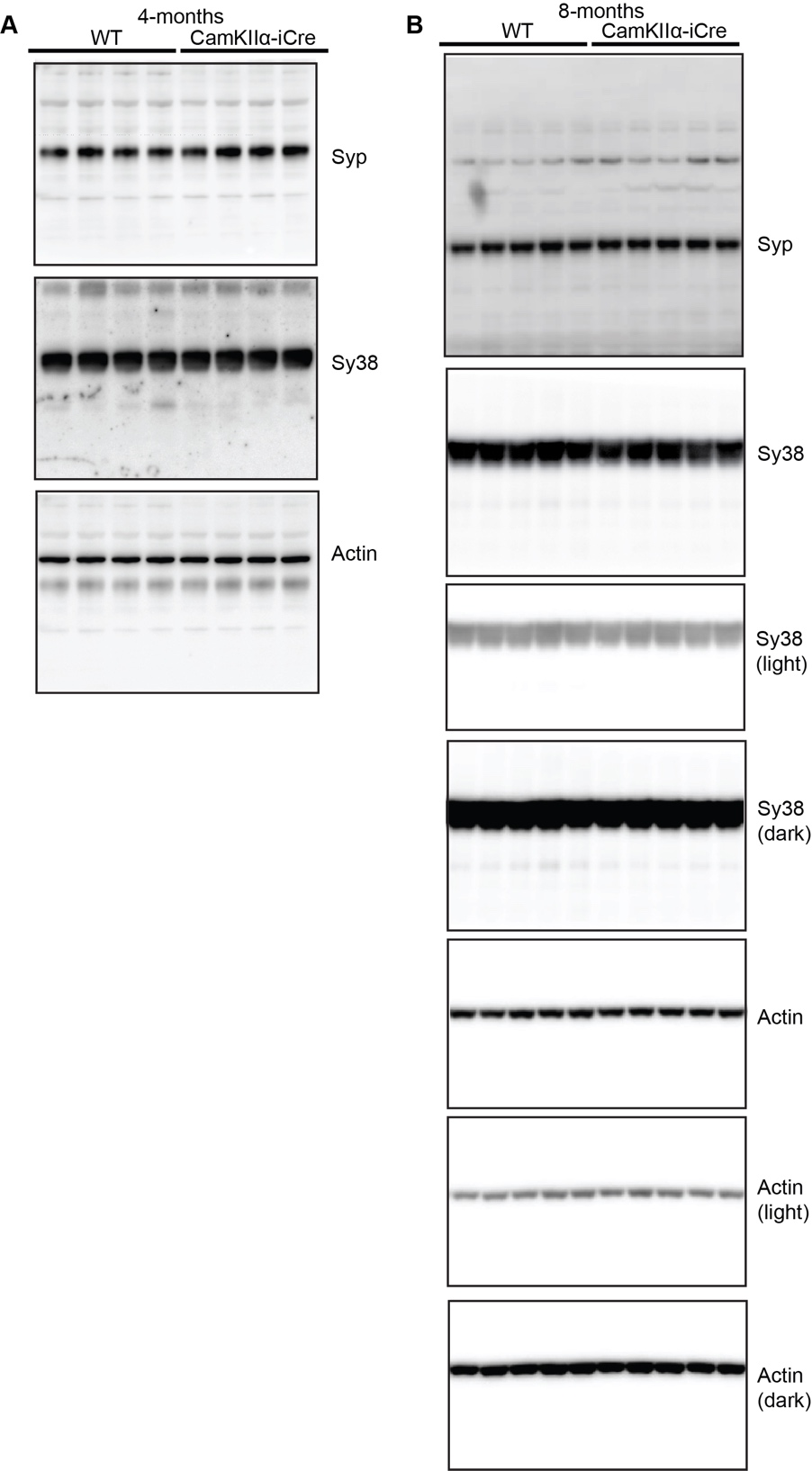


**Supplementary Figure S5**. **A)** Original immunoblots of 4-month-old mice shown in Fig. 4A. **B)** Original immunoblots of 8-month-old mice shown in Fig. 4A.


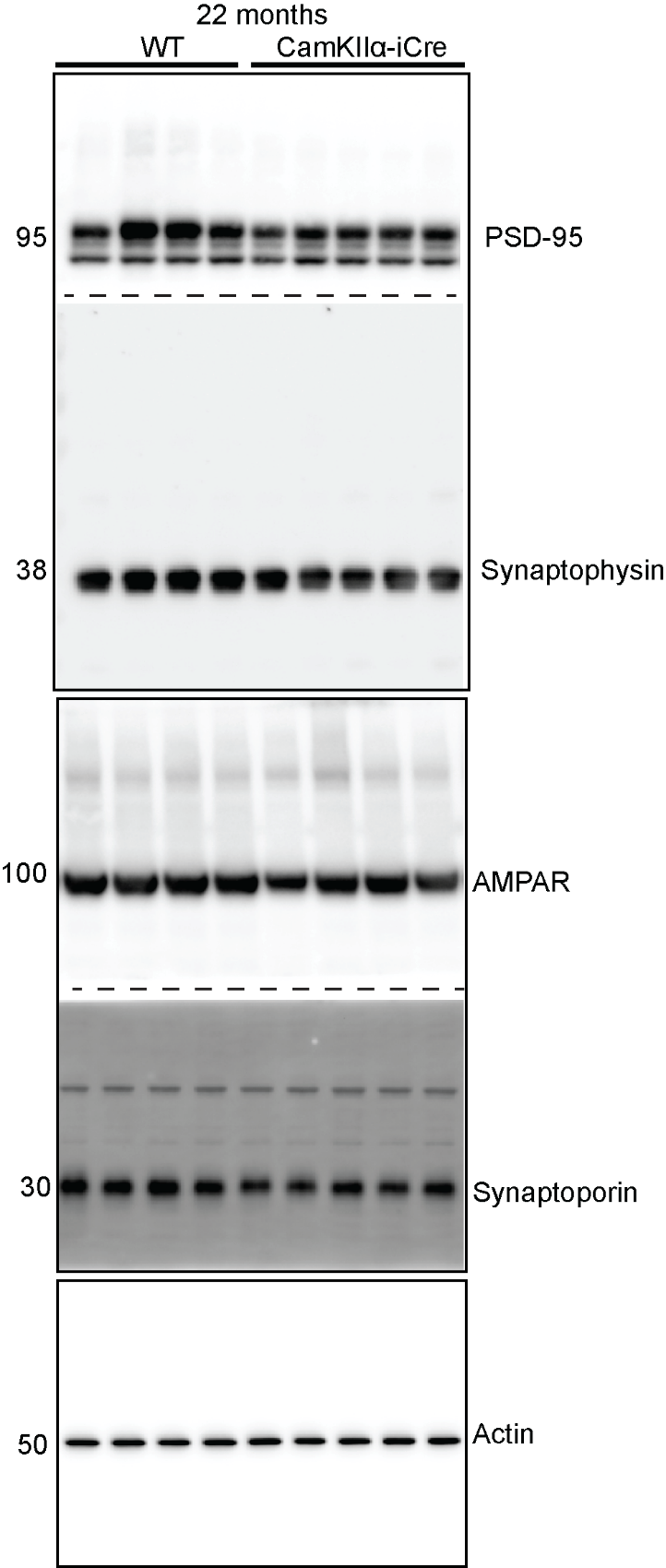


**Supplementary Figure S6**. **A)** Original immunoblots of 22-month-old mice shown in Fig. 5I. Dashed line indicates blots that were cut and probed with different antibodies.


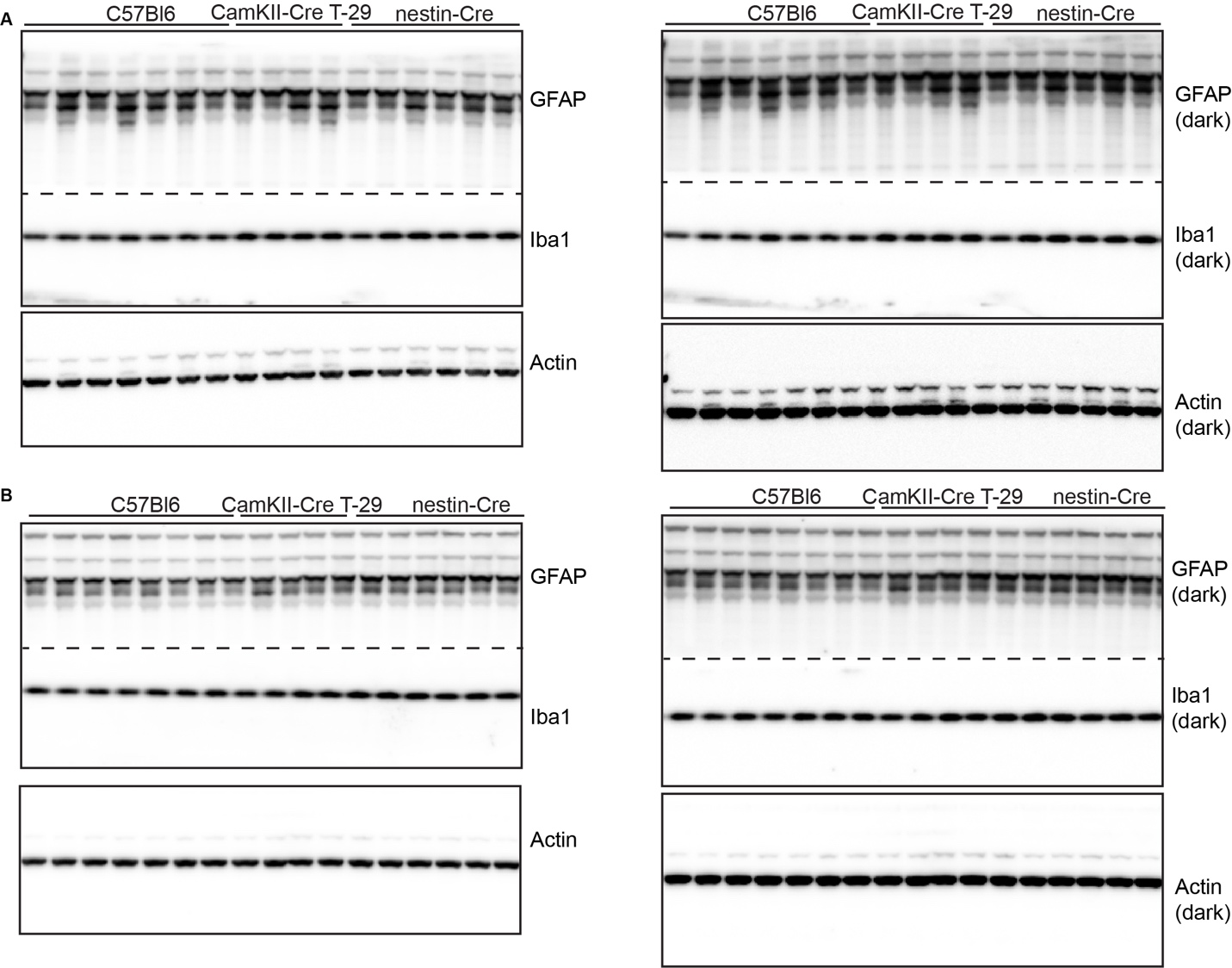


**Supplementary Figure S7**. **A)** Original immunoblots of 6B; an additional exposure is shown to the right. **B)** Original immunoblots of 6F; an additional exposure is shown to the right. Dashed line indicates blots that were cut and probed with different antibodies.

**
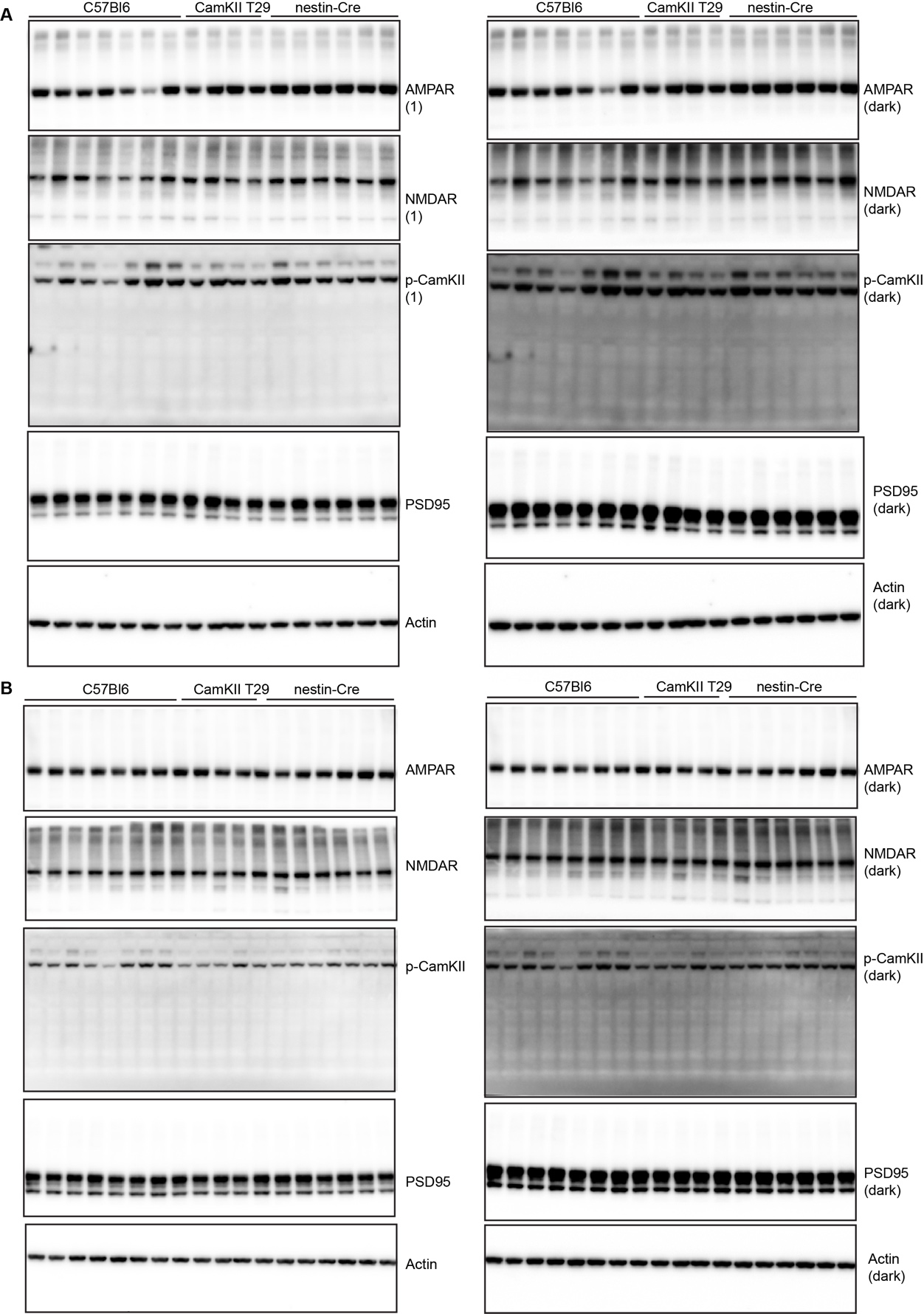
**

**Supplementary Figure S8**. **A)** Original immunoblots of 7A; an additional exposure is shown to the right. **B)** Original immunoblots of 7F; an additional exposure is shown to the right. Dashed line indicates blots that were cut and probed with different antibodies.
